# Identification of pyroptosis-related subtypes and comprehensive analysis of characteristics of the tumor microenvironment infiltration in clear cell renal cell carcinoma

**DOI:** 10.1038/s41598-023-43023-y

**Published:** 2023-09-25

**Authors:** Jiayi Zeng, Ping Zhu, Yanlin Tang, Changzheng Zhang, Chujin Ye, Shouyu Cheng, Kaiwen Tian, Bowen Yang, Weinan Zeng, Yanjun Liu, Zhiyong Xian, Yuming Yu

**Affiliations:** 1 Department of Urology, Guangdong Provincial People’s Hospital’s Nanhai Hospital, Foshan, China; 2 Department of Immunology, School of Basic Medical Science, Southern Medical University, Guangzhou, China; 3 Department of Urology, Guangdong Provincial People’s Hospital (Guangdong Academy of Medical Sciences), Southern Medical University, Guangzhou, China; 4 The Second School of Clinical Medicine, Southern Medical University, Guangzhou, China

**Keywords:** Cancer, Computational biology and bioinformatics

## Abstract

Pyroptosis is a kind of programmed cell death triggered by the inflammasome. Growing evidence has revealed the crucial utility of pyroptosis in tumors. However, the potential mechanism of pyroptosis in clear cell renal cell carcinoma (ccRCC) is still unclear. In this research, we systematically analyze the genetic and transcriptional alterations of pyroptosis-related genes (PRGs) in ccRCC, identify pyroptosis-related subtypes, analyze the clinical and microenvironmental differences among different subtypes, develop a corresponding prognostic model to predict the prognosis of patients, and interpret the effect of pyroptosis on ccRCC microenvironment. This study provides a new perspective for a comprehensive understanding of the role of pyroptosis in ccRCC and its impact on the immune microenvironment, and a reliable scoring system was established to predict patients’ prognosis.

## Introduction

As a common cause of death in patients with urological malignancies, Renal cell carcinoma (RCC) amount to about 3% of all malignant tumors in adults^1^. Worldwide, an estimated 403,000 new cases of RCC are diagnosed, and more than 175,000 people die from it each year^2^. Clear cell renal cell carcinoma (ccRCC) is considered to be the most common and deadly type, accounting for 70–80% of RCC^3^. Surgical resection is the main treatment for early ccRCC, while chemotherapy, targeting agents, and immunotherapy are the preferred treatments for advanced and metastatic ccRCC^4^. However, about 1/3 of patients with early RCC who receive surgical resection will have a recurrence or metastasis, and about 30% of the patients will have advanced or metastatic renal cell carcinoma when they first seek medical treatment^5^. Besides, ccRCC is insensitive to traditional chemotherapy and radiotherapy^6^, and the efficacy of targeted therapy and immunotherapy in advanced patients is still limited^7^, which greatly affects the prognosis of patients. Pieces of evidence have demonstrated that ccRCC has high immunogenicity^8^. Immunotherapy is becoming a effective new treatment strategy for ccRCC^9^. However, the response of ccRCC to immunotherapy is not satisfactory^10^. New therapeutic strategies need to be explored to improve the situation.

Pyroptosis is a kind of programmed cell death caused by inflammasome^11^. A strong inflammatory response caused by the release of cellular contents is a major feature of pyroptosis^12^. The occurrence of pyroptosis depends on the inflammatory caspases and the GSDMs protein family^13^. In terms of its mechanism, it is mediated by caspase-1 in canonical pathways and caspase-11 in non-canonical inflammasome pathways^14^. Recent studies have shown that pyroptosis participate in many human diseases, especially malignant tumors^15^. However, pyroptosis seems to play a dual role in tumor progression and therapeutic mechanisms^16^. Karki et al. believe that inflammatory factors released by pyroptosis can stimulate normal cells and cause them transform into tumor cells^17^. While pyroptosis can also inhibit tumor cells, making it a potential therapeutic target^12^.

New evidence also shows the close relationship between pyroptosis and tumor microenvironment (TME)^18^. TME was considered to be widely involved in the development and prognosis of tumors^19^. Its main components include immune cells, stromal cells, extracellular matrix molecules, cytokines, and chemokines^20^. The changes and interactions of various components are related to tumor progression and treatment outcome^21^. RCC is accompanied by obvious immune cell infiltration, including T cells, Monocytes, natural killer cells, dendritic cells, macrophages, etc.^22^. In most tumors, high infiltration levels of CD8 + T cells usually indicate a good prognosis. However, it is quite the opposite in ccRCC^23^, which indicates that the TME of ccRCC has a more complex regulation mechanism. Studies have shown that some cytokines and immunosuppressive cells can affect the TME of ccRCC, which leads to tumor immune escape^24^. In addition, as an important component of the TME, the tumor infiltrating immune cells (TIIC) can also serve as a predictive factor to help us predict the prognosis of patients^25^. However, the function and potential regulatory mechanism of pyroptosis in TME are still unclear. Thus, understanding the potential regulatory mechanisms of pyroptosis in ccRCC and the characteristics of immune cell infiltration in TME may provide a new direction for exploring the tumorigenesis mechanism of ccRCC and developing more effective immunotherapy strategies.

The purpose of the study is to analyze the genetic and transcriptional changes of pyroptosis-related genes (PRGs) in ccRCC, identify pyroptosis-related subtypes, analyze the clinical and microenvironmental differences among different subtypes, develop a corresponding prognostic model, and interpret the effect of pyroptosis on ccRCC microenvironment. In conclusion, our study provides a new perspective for comprehensive understanding the function and potential regulatory mechanism of pyroptosis in ccRCC and its impacts on the immune microenvironment, and a reliable scoring system was developed to assess the prognosis of patients.

## Methods and materials

### Data source

We downloaded the RNA transcriptome data of 539 ccRCC samples and 72 matched normal kidney samples from the TCGA database (https://portal.gdc.cancer.gov/). Patients’ characteristics were also obtained from the TCGA database. All the data is used for differential expression analysis. There are 491 ccRCC samples with complete data and the survival time > 3 months in the TCGA dataset. The 491 samples were stochastically divided into training cohort (n = 197) and testing cohort (n = 294) in a ratio of 4:6. A prognostic model was constructed in training cohort, testing cohort and TCGA entire cohort were as validation datasets to validate the performance of the model. Besides, the information of the E-MTAB-1980 cohort was obtained from the ArrayExpress database (https://www.ebi.ac.uk/biostudies/arrayexpress/), which was used as the external validation dataset.

### Identification of differentially expressed PRGs

We retrieved 52 pyroptosis-related genes (PRGs) from the MSigDB database (REACTOME_PYROPTOSIS) (http://www.broad.mit.edu/gsea/msigdb/) and the previous publication^26^. The differentially expressed PRGs between normal kidney tissues and tumor tissues was identified by the R package "limma" with the set threshold was |log2FC|> 0 and false discovery rate (FDR) < 0.05. Then, the protein–protein interaction (PPI) networks of these PRGs was constructed through the Search Tool for the Retrieval of Interacting Genes (STRING) to further explore the interactions of these PRGs with the minimum required interaction score was set at 0.9 (the highest confidence) and removed the isolated genes. At the same time, the scoring data of nodes were downloaded and the hub genes were identified by weighting algorithm in the Cytoscape. Next, univariate Cox regression analysis was run to judge the prognostic value of PRGs and *P* < 0.05 is considered to be a meaningful result. The R package "igraph", "reshape2" and "RColorBrewer" were used to construct a prognostic network.

### Somatic mutation and copy number variation

The somatic mutation of ccRCC was obtained from the TCGA database and the copy number variation (CNV) was gained from the Xena Functional Genomics Explorer (https://xenabrowser.net/datapages/). The R package "maftools" was performed to analyze the mutation frequency and draw waterfall map of differentially expressed PRGs. Next, the CNV changes of 37 PRGs on 23 human chromosomes were located by the R package "RCircos".

### Consensus clustering analysis of PRGs

We executed a consensus clustering analysis on patients via the R package “ConsensusClusterPlus” to identify different pyroptosis-related subtypes. We think it is the best type when the subtype number k = 3. The principal component analysis (PCA) was subsequently executed to assess the gene expression profile in different subtypes. Then, the gene set variation analysis (GSVA) was performed to explore the biological function of PRGs in different pyroptosis-related subtypes by the signature gene set (c2.cp.kegg.v7.2) obtained from the MSigDB database (http://www.broad.mit.edu/gsea/msigdb/).

### The relationship between pyroptosis-related subtypes and clinical characteristics and prognosis of ccRCC

In order to evaluate the potential value of three pyroptosis-related subtypes identified from unsupervised cluster analysis, we compared the prognosis and clinical characteristics (including gender, age, survival status, TNM stage, AJCC stage, and grade) among different subtypes. The Kaplan–Meier curve generated by the R package "survival" and "survminer" was used to compare the differences in overall survival (OS) and progression free survival (PFS) among three subtypes.

### The immune characteristics of pyroptosis-related subtypes

The ESTIMATE algorithm was run to calculated the immune and stromal scores of each patient to explore the TME differences in different subtypes. The CIBERSORT algorithm was executed to estimate the fractions of 22 human immune cell of every ccRCC sample so that we can assess the levels of immune cell infiltration. Moreover, we also analyzed the expression levels of PD-1 and PD-L1 among different subtypes.

### Identification of DEGs and functional analysis in different pyroptosis-related subtypes

The R package “limma” was used to identify of differentially expressed genes(DEGs) among different subtypes with the threshold |log2FC|> 0 and the adjusted P-value < 0.001. To further explore the functional pathways and related potential functions of these DEGs, we executed the functional enrichment analysis by the R package "clusterprofiler", including Gene Ontology (GO) and Kyoto Encyclopedia of Genes and Genomes (KEGG).

### Construction and validation of a pyroptosis-related gene prognostic PRGP_score

We firstly removed lncRNAs from the previously obtained DEGs and performed the univariate Cox regression analysis on the remaining DEGs to identify prognosis-related genes. Then, the "limma" package was executed to identify the differential expression of these prognostic-relevant genes between normal kidney tissues and tumor tissues, with the threshold set as the |log2FC|> 1 and the adjusted P-value < 0. 001. The LASSO Cox regression analysis was conducted on the resulting genes to further reduce the number of genes. Next, multivariate Cox regression analysis was executed and 4 genes (ENGASE, LRFN1, CDKL2, IFI44) were finally selected to construct the PRGP_score in the training cohort. The performance of the PRGP_score was validated in the testing cohort, the TCGA entire cohort, and the E-MTAB-1980 cohort.

The PRGP_score was calculated by the following formula:

$${\text{PRGP}}\_{\text{score}} = \Sigma \left( {{\text{Xi}}*{\text{Yi}}} \right)$$

where Xi and Yi represented the risk coefficient and expression levels of each gene, respectively. On the basis of the median value of the PRGP_score, patients were divided into low- and high-risk group. The Kaplan–Meier curves was used to compare the OS difference in the two groups. The efficiency of the scoring model was evaluated according to the corresponding ROC curve. Then, we performed the PCA to assess whether the model could distinguish patients into different groups via the R package "ggplot2". The same analysis were performed in both internal and external datasets to judge the effectiveness of the model.

### Clinical correlation, independent prognostic analysis and stratification analysis of PRGP_score

The chi-square test was conducted to estimate the correlation between PRGP_score and these clinical features. Univariate and multivariate Cox regression analyses were executed on clinical characteristics and PRGP_score To confirm whether PRGP_score could be independent of other clinical characteristics. Furthermore, we also executed a stratification analysis to judge whether the scoring model maintains its good predictive power in different subgroups.

### Transcriptome sequencing

We collected 18 tumor samples and 6 paracancerous samples from 6 ccRCC patients who treated in Guangdong Provincial people's Hospital. According to the manufacturer's instructions, each case of ccRCC tissue and matched paracancerous samples were sequenced at the end of the pair on the NovaSeq 6000 high-throughput sequencing platform (Illumina,USA). After removing the sequencing reads containing aptamer sequences and low-quality reads and low-quality bases, HISAT2 (v2.1.1) is used to align the high-quality paired reads with the human genome GRCh38 and generate the BAM file. Using samtools (v1.15.1) tool to sort the BAM files, and then tool Subread (v2.0.1) to count. The original count of transcripts per gene is converted to the fragments per kilobase million (FPKM), which makes the gene expression between samples more directly comparable.

### TME and immune checkpoints

The ESTIMATE algorithm was used to estimate the immune score and stromal score of samples so that we can investigate the differences in TME among the low- and high-risk group. Then, the correlation coefficient was calculated by the Spearman correlation analysis. In addition, the infiltration fractions of 22 human immune cells in two groups were estimated via the CIBERSORT algorithm and the difference in infiltration were showed by radar map. Furthermore, we obtained the Tumor Immune Dysfunction and Exclusion (TIDE) score of ccRCC patients from the TIDE website (http://tide.dfci.harvard.edu/). The differences of TIDE scores in two groups were compared to further forecast the potential efficacy of immunotherapy in different groups. Finally, the expression levels of common immune checkpoint genes were also investigated among two groups.

### Mutation and drug susceptibility analysis

The mutation distribution between low- and high-risk group were visualized via the waterfall plot generated by the "maftools" packet in R. We extracted the tumor mutational burden (TMB) of each ccRCC patient. They were divided into low- and high-TMB group according to the median value of TMB, and the survival differences of two groups was showed by Kaplan–Meier curves. Then, the correlation of PRGP_score with TMB was calculated through the Spearman correlation analysis, and the TMB differences in two groups were compared. Finally, we calculated the semi-inhibitory concentration (IC50) values of some anticancer drugs using the "pRRophetic" package to compare the sensitivity of two groups to common drugs.

### Development of a nomogram

We developed a nomogram via the "rms" package based on PRGP_scores and some clinical information with independent prognostic value to predict the probability of 1-, 3- and 5-year OS. The predictive accuracy of the nomogram was assessed by the ROC curves. Calibration plots of the nomogram were used to depict the predictive value between the predicted survival events and the virtually observed outcomes, and the 45°slash represents the best prediction performance.

### Statistical analyses

All the above statistical analyses and R packages were executed in the R 4.0.4 versions (http://www.R-project.org). All the results of statistical analyses are two-way, and P-value < 0.05 is considered to be meaningful result.

## Results

### Genetic and transcriptional changes of PRGs in ccRCC

**Figure 1:**
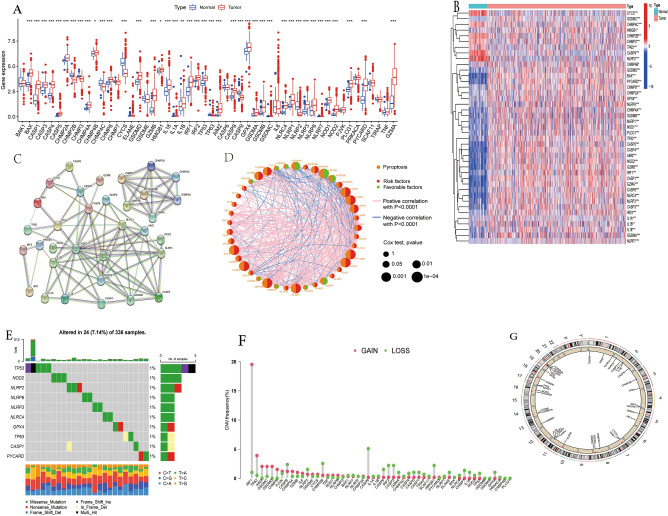
Genetic and transcriptional changes of PRGs in ccRCC. (**A**) The mNRA levels of 52 PRGs in the TCGA dataset. (**B**) The expression heatmap of 41 PRGs of the TCGA dataset. (**C**) The result of protein–protein interaction (PPI) analysis of PRGs. (**D**) The pyroptosis gene prognosis network. (**E**) The somatic mutations Top10 of PRGs in ccRCC. (**F**) The copy number variations (CNV) of 52 PRGs. (**G**) The changes of 37 PRGs with characteristics of CNV on their respective chromosomes. *P < 0.05, **P < 0.01, ***P < 0.001.

Comparing the expression levels of 52 PRGs in ccRCC samples and normal kidney samples, 41 DEGs (P < 0.05) were identified. Specifically, 32 genes were up-regulated (GZMA, AIM2, CASP5, GZMB, NOD2, NLRP7, GSDMC, PYCARD, GSDMB, NLRC4, CASP1, NLRP6, GSDMA, NLRP3, CASP4, NLRP1, BAX, IRF1, CHMP4A, L18, CASP8, GSDMD, NOD1, PLCG1, CASP3, GPX4, TP53, CHMP6, CHMP2A, IRF2, CHMP4B, HMGB1) and 9 genes were down-regulated (IL1B, CHMP3, CHMP4C, CHMP2B, IL1A, CASP9, CYCS, TP63, NLRP2) in ccRCC tissues. The specific expression level and expression heatmap of these genes are shown in Fig. 1A,B. As shown in Fig. 1C, the PPI network shows the possible interactions of these PRGs, CASP1, PYCARD, NLRP3, NLRC4, AIM2, GSDMD, NLRP1, and CASP5 were considered to be the hub genes. As shown in Fig. 1D, pyroptosis gene prognosis network distinctly reveals the correlation of these PRGs and their relationship with prognosis. Lines between circles indicate correlations between pyroptotic genes, blue indicates negative correlations, pink indicates positive correlations. The right half of the circle represents the risk effect of the gene on the patient, with green representing protective factors and red representing risk factors. The size of the circle represents the p value.

At the genetic level, the Top 10 somatic mutations of PRGs in ccRCC are shown in Fig. 1E. Of the 336 ccRCC samples, only 24 samples(7.14%) had mutations in the PRGs, and the overall mutation rate was low. We found that TP53 had the highest mutation frequency, followed by NOD2. Missense mutation is the most common mutation type, followed by nonsense mutation. Next, we explored the CNV of all PRGs, and found CNV in 37 PRGs. IRF1, TP63, AIM2, GSDMC, and GSDMD had widespread CNV increases, while CASP9, CHMP2B, IRF2, CASP3, and HMGB1 had CNV loss (Fig. 1F). The changes of 37 PRGs with characteristics of CNV on their respective chromosomes are shown in Fig. 1G. Compared with normal tissues, the expression levels of PRGs with increased CNV such as IRF1, AIM2 and GSDMD, were significantly increased in ccRCC samples. While genes with CNV loss such as CASP9 and CHMP2B, their expression levels were significantly decreased in ccRCC samples, indicating that CNV may positively related to the expression levels of PRGs. We speculate that there may be a regulatory relationship between CNV and the expression of PRGs.

However, we also noticed that TP63 with increased CNV, had lower mRNA levels in ccRCC samples. While for some genes with CNV loss, their mRNA levels were not significantly different in normal kidney tissue and ccRCC samples. Therefore, CNV may not be the only factor in regulating gene expression. The results of our analysis show that the genetic and transcriptional alterations of PRGs between normal kidney tissues and ccRCC samples were momentous difference, and PRGs may have a potential function in the occurrence and progression of ccRCC.

### Identification of pyroptosis-related subtypes in ccRCC

**Figure 2:**
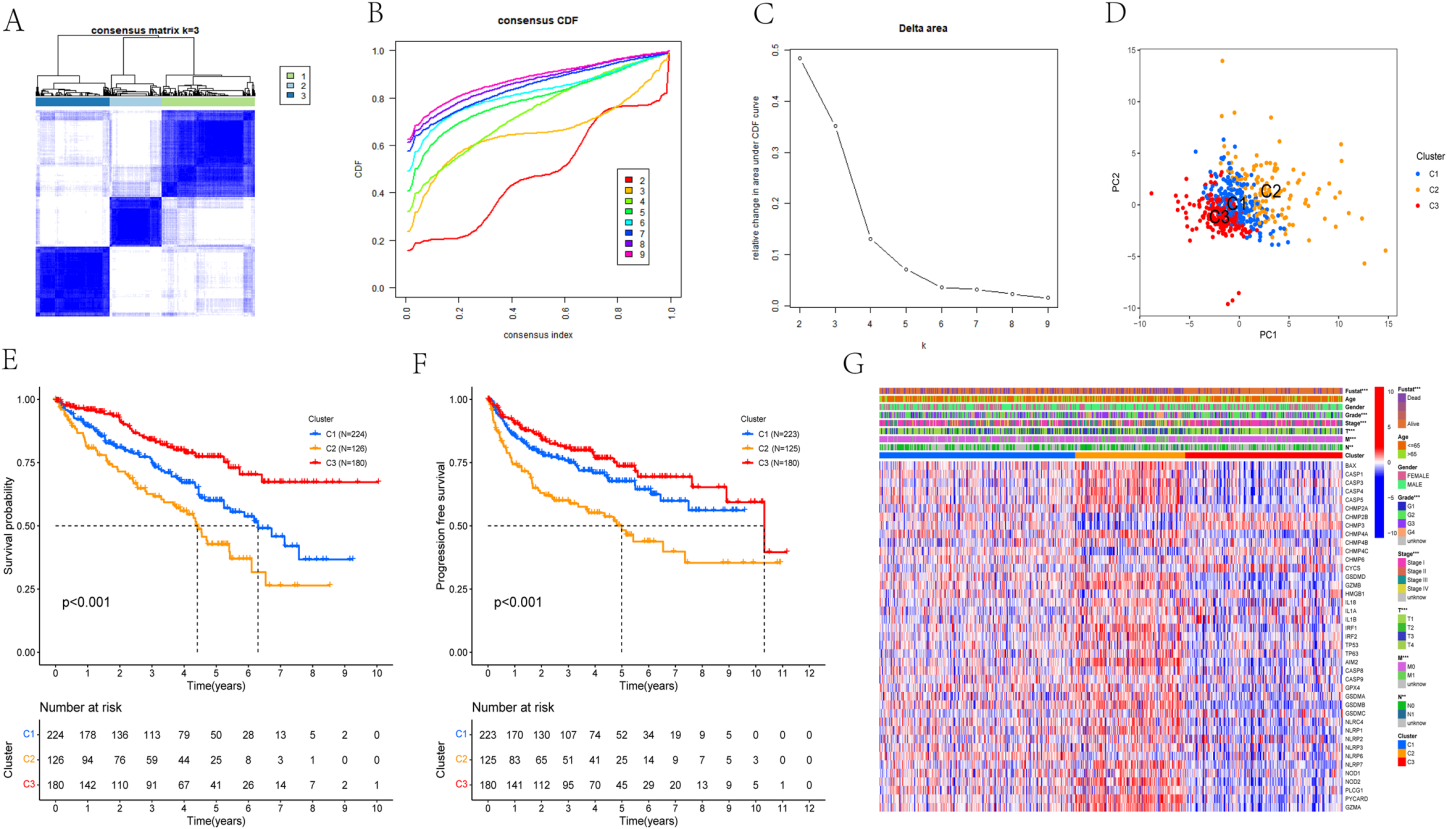
Identification of pyroptosis-related subtypes. (**A**–**C**) Consensus matrix heatmap defining three clusters (k = 3) and their correlation area and cumulative distribution function (CDF) curve. (**D**) PCA analysis reveals the obvious differences in gene expression profiles among three subtypes. (**E**,**F**) Kaplan–Meier curves of OS and PFS for patients with ccRCC among three subtypes. (**G**) Differences in clinicopathologic features and the expression levels of PRGs among three subtypes.

In order to explore the relationship between these 41 differentially expressed PRGs and ccRCC subtypes, consensus clustering analysis was executed on 530 ccRCC patients of the TCGA cohort and three pyroptosis-related subtypes were identified named C1, C2 and C3 (Fig. 2A–C). PCA was then performed to evaluate the stability of clustering. As shown in Fig. 2D, distinct differences in gene expression profiles among the three subtypes were observed. We further analyzed the prognosis and clinical characteristics in different subtypes. Obvious differences in OS and PFS was found among the three subtypes via Kaplan–Meier curves. Among them, the subtype C3 had the best prognosis, subtype C1 followed, and subtype C2 had the worst prognosis (Fig. 2E,F). Besides, there are obvious differences in the expression of PRGs and clinical characteristics among different subtypes. Strong differences in survival status, grade, AJCC stage, and TNM stage were found among three subtypes, while no statistical differences in age and gender (Fig. 2G). Specifically, the subtype C2 was associated with higher grade, AJCC stage, and TNM stage, and the overall expression level of PRGs of was higher with the highest proportion of deaths compared to the other two subtypes.

### Differences in TME among different subtypes

**Figure 3:**
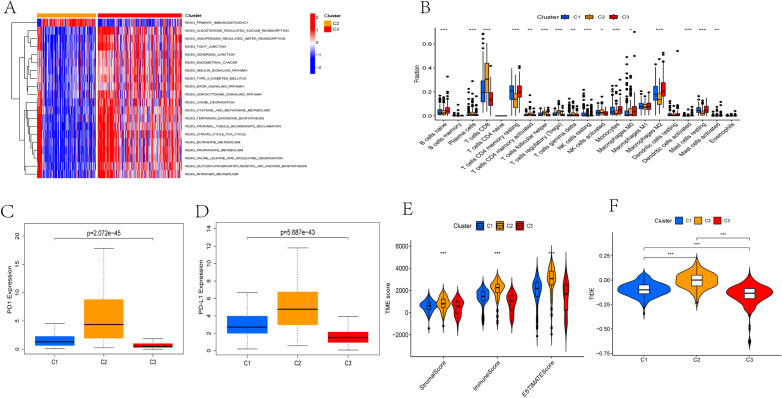
Differences in TME among the three pyroptosis-related subtypes. (**A**) The differences of GSVA between subtypes C2 and C3. (**B**) The infiltration scores of immune cells among three subtypes. (**C**,**D**) The expression levels of PD-1 and PD-L1 among three subtypes. (**E**) The TME score of patients among the three subtypes. (**F**) The TIDE score of patients among the three subtypes. *P < 0.05, **P < 0.01, ***P < 0.001.

The Gene set variation analysis (GSVA) showed that subtype C2 was significantly enriched in immunedeficiency, while subtype C3 was enriched in metabolism, such as insulin signal pathway, adipocytokine signal pathway, TCA cycle, amino acid degradation, and so on (Fig. 3A). As shown in Fig. 3B, most immune cells show differences among three subtypes. Among them, plasma cells, T cells CD8, T cells CD4 memory activated, T cells follicular helper, T cells regulatory (Tregs), T cells gamma delta, and NK cells activated have the highest proportion in the subtype C2, while B cells naive, Monocytes, Macrophages M0, Macrophages M2 and Mast cells resting have the highest proportion in the subtype C3. Then, the expression levels of 2 important immune checkpoint genes were also compared among three subtypes. As shown in Fig. 3C,D, PD-1 and PD-L1 were highly expressed in subtype C2 while low in the subtype C3. Next, we further used the ESTIMATE algorithm to compute the TME score of each patient among different subtypes. The TME score of subtype C2 was the highest, suggesting the relative high content of stromal cells and immune cells in subtype C2 compared to other two subtypes (Fig. 3E). Finally, the TIDE score of different subtypes was used to judge the potential efficacy of immunotherapy for ccRCC patients. The higher TIDE score, the higher potential for immune escape, suggesting that patients are less likely to benefit from ICIs treatment. The results revealed that the subtype C2 had the highest TIDE score, while the subtype C1 had the lowest TIDE score (Fig. 3F), which may explain their prognosis.

### Identification of gene subtypes based on DEGs

**Figure 4:**
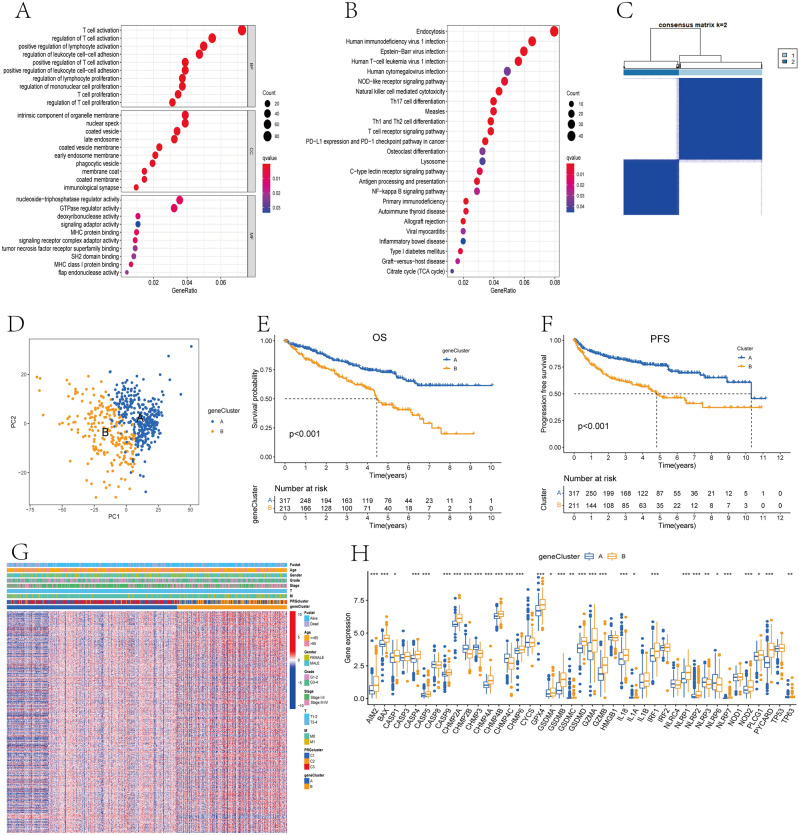
Identification of different gene subtypes. (**A**,**B**) GO and KEGG enrichment analyses of DEGs among three pyroptosis-related subtypes. (**C**) Consensus cluster analysis defining two gene subtypes. (**D**) PCA analysis shows obvious difference in gene expression profiles among two gene subtypes. (**E**,**F**) Kaplan–Meier curves of OS (**E**) and PFS (**F**) for patients with ccRCC between two gene subtypes. (**G**) Correlation between clinical characteristics and two gene subtypes. (**H**) Difference in the expression levels of PRGs among two gene subtypes. *P < 0.05, **P < 0.01, ***P < 0.001.

In order to explore the differences in potential biological functions among different pyroptosis-related subtypes, we used the "limma" package to identify DEGs among them, with the threshold set as |log2FC|> 1 and adjusted p-value < 0.001. 2041 DEGs were identified and functional enrichment analysis was executed on them. GO enrichment analysis revealed that immune-related biological processes were strongly enriched, such as T cell activation, proliferation and regulation, lymphocyte activation, and proliferation regulation (Fig. 4A). KEGG enrichment analysis showed that immune and cancer-related pathways were obviously enriched, such as T cell receptor signaling pathway, NOD-like receptor signaling pathway, PD-L1 expression and PD-1 checkpoint pathway in cancer, and NF-kappa B signaling pathway (Fig. 4B). It shows that PRGs may play an vital role in the immune regulation of TME in ccRCC.

In order to further explore the potential value of these DEGs, we removed the lncRNAs and obtained 1310 genes with protein-coding functions, and performed an univariate Cox regression analysis to screen out 790 genes related to prognosis. Then, patients were classified into gene subtype A and B using the consensus cluster analysis (Fig. 4C). PCA showed that these two gene subtypes were very ideal (Fig. 4D). Kaplan–Meier curves revealed the evident difference in OS and PFS between two gene subtypes, and patients in the gene subtype A group had a good prognosis (Fig. 4E,F). Furthermore, the gene subtype B group was related to higher grade, AJCC stage, and TNM stage (Fig. 4G). And momentous difference in the expression of PRGs was also found in two subtypes (Fig. 4H), indicating PRGs may participate in the occurrence and progression of ccRCC.

### Construction of the PRGP_score

**Figure 5:**
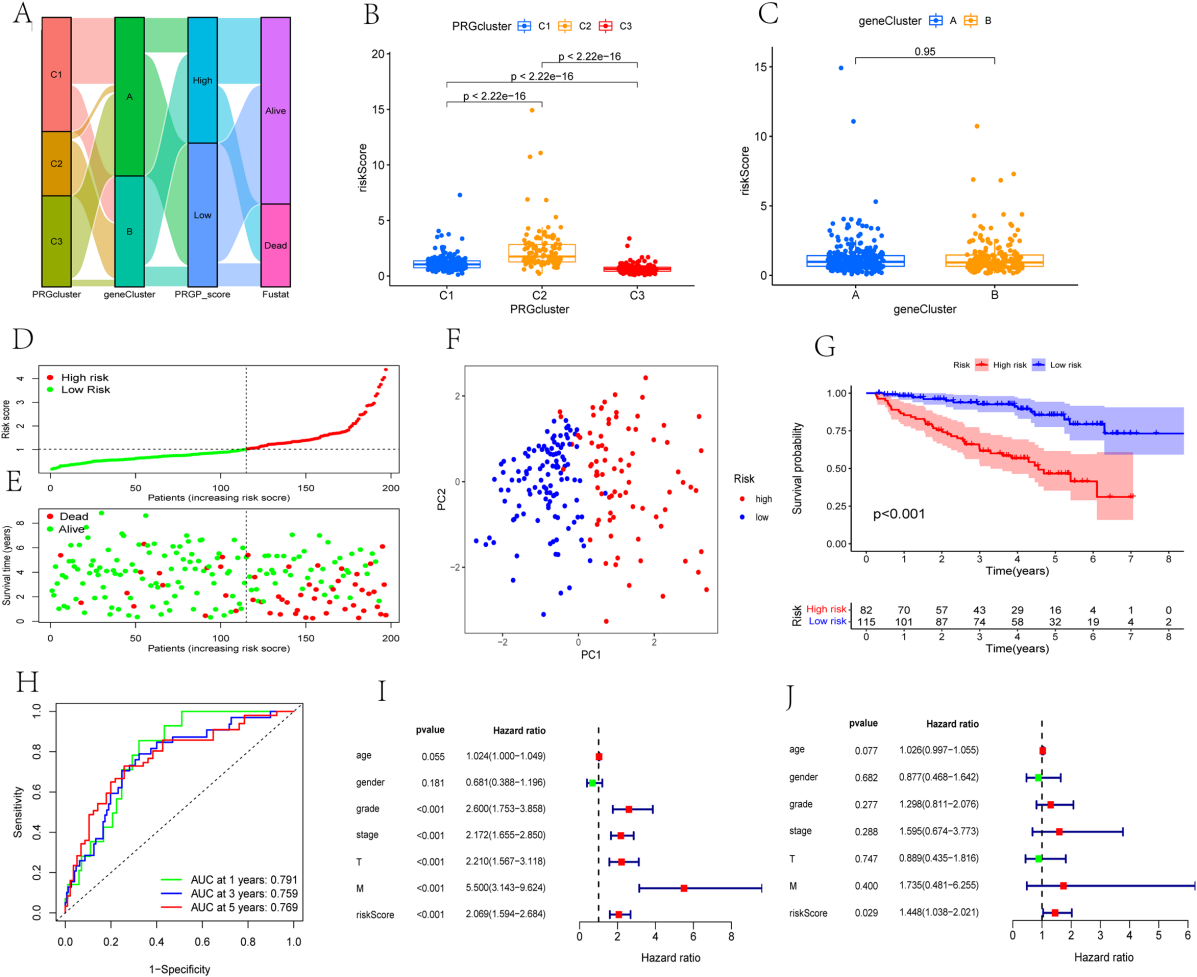
Construction of the PRGP_score in the training cohort. (**A**) Sankey diagram shows the distribution of patients in three pyroptosis-related subtypes, two gene subtypes, and two PRGP_score groups. (**B**) Differences in PRGP_score among the three pyroptosis-related subtypes. (**C**) Difference in PRGP_score among two gene subtypes. (**D**,**E**) The risk distribution curve and survival status of patients. (**F**) PCA shows an evident distinction between two groups. (**G**) Kaplan–Meier curves of OS for patients with ccRCC between low- and high-risk group. (**H**) The ROC curves of the PRGP_score to predict the survival rates of 1-, 3-, and 5-year. (**I**,**J**) Univariate and multivariate Cox regression analysis of PRGP_score and clinicopathologic features.

Based on the DEGs between different pyroptosis-related subtypes obtained earlier, we constructed the PRGP_score. Sankey diagram shows the distribution of patients in three pyroptosis-related subtypes, two gene subtypes, and two PRGP_score groups (Fig. 5A). First, we identified 790 genes associated with prognosis in the previously obtained DEGs. The "limma" package was run to identify the differential expression of these 790 genes in normal and tumor tissues. The threshold was set as |log2FC|> 1 and adjusted p-value < 0.001, and 221 genes were identified. Then, LASSO Cox regression analysis was executed to further reduce the number of genes. Finally,4 genes were selected to construct PRGP_score in the training cohort through multivariate Cox regression analysis (Fig. S1). Among them, ENGASE, LRFN1, and IFI44 are risk genes, while CDKL2 is a protective gene (Fig. S2).

$$\begin{aligned} {\text{PRGP}}\_{\text{score}} & = \left( {0.{1}0{54}*{\text{mRNA levels of ENGASE}}} \right) + \left( {0.{3891}*{\text{mRNA levels of LRFN1}}} \right) \\ & \quad + \left( {0.0{18}0*{\text{mRNA levels of IFI44}}} \right)\, + \,( - 0.{4479}*{\text{mRNA levels of CDKL2}}) \\ \end{aligned}$$

Evidential difference in PRGP_score were found among three pyroptosis-related subtypes. The subtype C2 had the highest PRGP_score, and the subtype C3 had the lowest PRGP_score, which indicates that a high PRGP_score may be associated with immunodeficiency and microenvironmental suppression, while a low PRGP_score is related to substance metabolism (Fig. 5B). However, statistical difference in PRGP_score was not observed among two gene subtypes (Fig. 5C). We divided patients into a high- and low-risk group in the training cohort according to the median value of PRGP_score. The risk distribution curve and survival status demonstrated that the survival time of patients decreased and the death toll increased with the increase of PRGP_scores (Fig. 5D,E). PCA shows a obvious distinction between two groups (Fig. 5F). Kaplan–Meier curve showed distinct difference in OS between two groups, and low-risk group had a good prognosis (Fig. 5G). The AUC values of 1-, 3-, and 5-year survival rates of PRGP_score were 0.791, 0.759 and 0.769, respectively, which shows a satisfactory prediction efficiency (Fig. 5H). In addition, univariate and multivariate Cox regression analysis demonstrated that the PRGP_score was an independent prognostic factor for ccRCC patients (Fig. 5I,J).

### Internal and external validation of the PRGP_score

**Figure 6:**
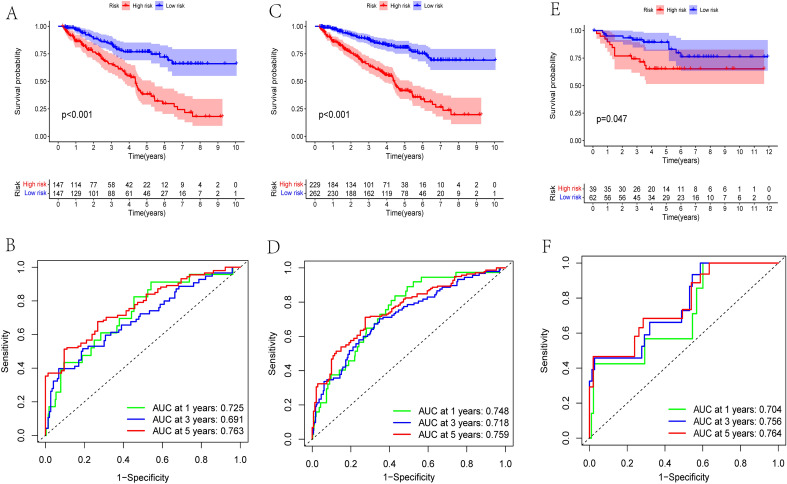
Internal and external validation of the PRGP_score. (**A**) Kaplan–Meier curves between two groups in testing cohort. (**B**) The ROC curves of the PRGP_score to predict the survival rates of 1-, 3-, and 5-year in testing cohort. (**C**) Kaplan–Meier curves between two groups in the TCGA entire cohort. (**D**) The ROC curves of the PRGP_score to predict the survival rates of 1-, 3-, and 5-year in TCGA entire cohort. (**E**) Kaplan–Meier curves between two groups in E-MTAB-1980 external cohort. (**F**) The ROC curves of the PRGP_score to predict the survival rates of 1-, 3-, and 5-year in E-MTAB-1980 external cohort.

In order to validate the prognostic performance of PRGP_score in different patients, we verified it in internal and external cohorts. In testing cohort, Kaplan–Meier analysis showed a worse prognosis in high-risk group (Fig. 6A). The AUC values of the PRGP_score to predict OS of 1-, 3-, and 5-year were 0.725, 0.691 and 0.763, respectively (Fig. 6B). In TCGA entire cohort, a worse prognosis was also observed in high-risk group (Fig. 6C). The PRGP_score still shows good prediction performance. The 1-, 3-, and 5-year OS of PRGP_score were represented by AUC values of 0.748, 0.718 and 0.759, respectively (Fig. 6D). Furthermore, we conducted external validation in the E-MTAB-1980 cohort to further evaluate the predictive reliability and accuracy of PRGP_score. Similar results were obtained in E-MTAB-1980 external cohort, high-risk group had a worse survival compared to low-risk group (Fig. 6E). The AUC values of the PRGP_score to predict OS of 1-, 3-, and 5-year were 0.704, 0.756 and 0.764, respectively (Fig. 6F). It shows that PRGP_score has good stability and prediction efficiency in different cohorts. The risk distribution curves, survival status, PCA analysis results, and results of univariate and multivariate Cox prognostic analysis of the three cohorts are presented in Fig. S3.

### Verification of the expression levels of 4 genes constructing PRGP_score

The transcription levels of 4 genes were verified by transcriptome sequencing of the collected 18 tumor samples and 6 paracancerous samples (Fig. S4). The results of sequencing were similar to TCGA database, the mRNA levels of ENGASE, LRFN1, and IFI44 were obvious higher in ccRCC tissues than that in corresponding paracancerous tissues. The expression of CDKL2 was consistent with our expectations, its mRNA levels was lower in ccRCC than that in paracancerous tissues.

### Clinical correlation analysis and stratified analysis of PRGP_score

In order to further explore the effects of PRGP_score on clinical features, we assessed the relationship between PRGP_score and different clinicopathological features (including age, gender, survival status, T stage, M stage, grade, and AJCC stage) in TCGA entire cohort. The proportion of deaths in high-risk group increased strongly and was associated with worse T stage, M stage, grade, and AJCC stage (Fig. S5). Then, we processed a stratified analysis to further judge the applicability of PRGP_score in different clinical subgroups. In all subgroups, high-risk group were associated with worse OS, revealing the satisfactory application value of PRGP_score (Fig. S6).

### TME and immune checkpoints between low- and high-risk group

**Figure 7:**
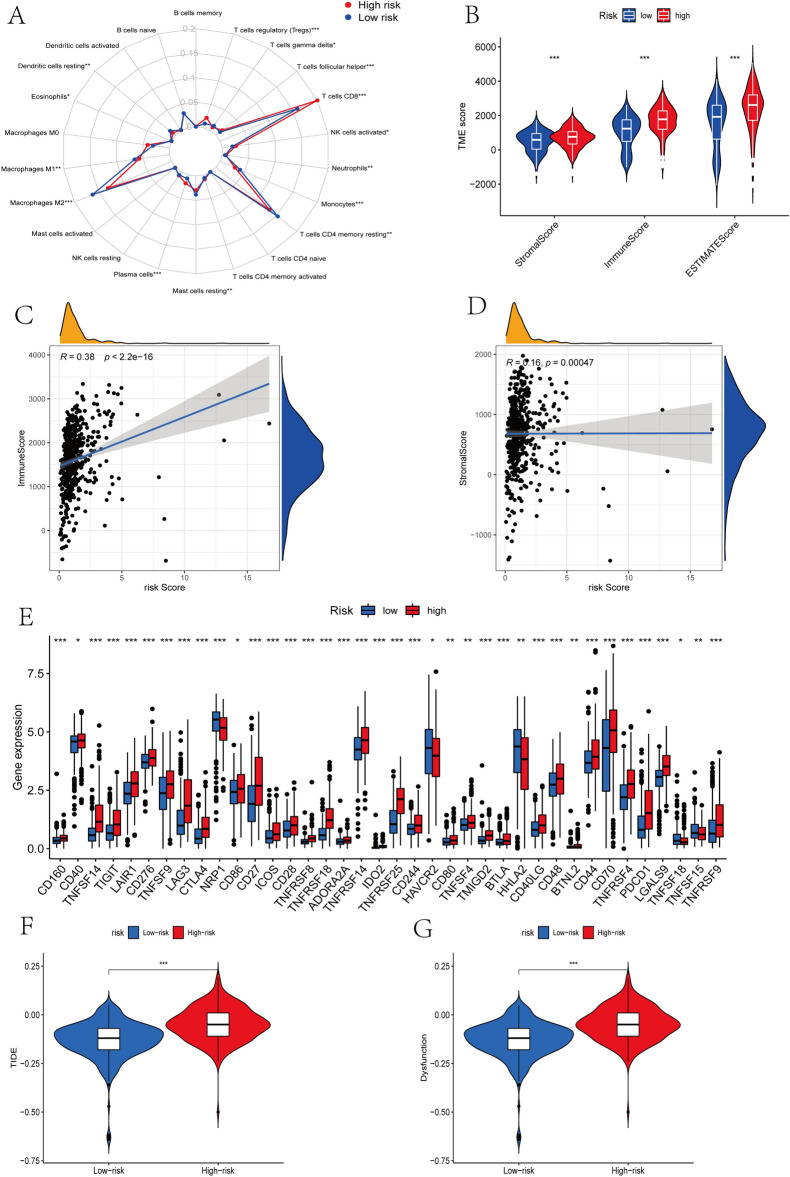
Evaluation of the TME and immune checkpoints between high- and low-risk group. (**A**) Radar map shows the difference of immune cell infiltration abundance between two groups. (**B**) The ESTIMATE assessment results show the differences of TME scores between two groups. (**C**,**D**) Correlations between PRGP_score and both immune and stromal scores. (**E**) The mRNA levels of immune checkpoints in two groups. (**F**,**G**) The differences of TIDE score between two groups.

The radar map shows that high-risk group had a higher cell infiltration level of Plasma cells, NK cells activated, T cells CD8, T cells regulatory (Tregs) and T cells follicular helper, while low-risk group had a higher cell infiltration level of Dendritic cells resting, Macrophages M1, Macrophages M2, T cells CD4 memory resting, T cells gamma delta, Monocytes, Neutrophils and Mast cells resting (Fig. 7A). Spearman correlation analysis demonstrated that PRGP_score was positively correlated with T cells follicular helper, T cells CD8, T cells CD4 memory activated, Plasma cells and Tregs, and negatively correlated with Monocytes, Macrophages M2, Mast cells resting, Macrophages M1, Neutrophils, T cells CD4 memory resting and Dendritic cells resting (Fig. S7). The ESTIMATE assessment results revealed momentous differences in TME scores between two groups, patients in high-risk group having a higher immune and stromal scores (Fig. 7B). And as PRGP_score increases, so do immune and stromal scores (Fig. 7C,D). Besides, we analyzed the relationship between some immune checkpoints and PRGP_score. The mRNA levels of 38 immune checkpoint genes were different between two groups, including PD-1, CTLA-4, LAG3, and so on (Fig. 7E). Finally, we also assessed the potential efficacy of immunotherapy in high- and low-risk group by the TIDE score. Compared to low-risk group, both the TIDE score and T-cell dysfunction score were higher in high-risk group (Fig. 7F,G), suggesting that high-risk group patients may benefit less from ICIs therapy, and T-cell dysfunction may be an important reason.

### Mutation and drug sensitivity analysis

**Figure 8:**
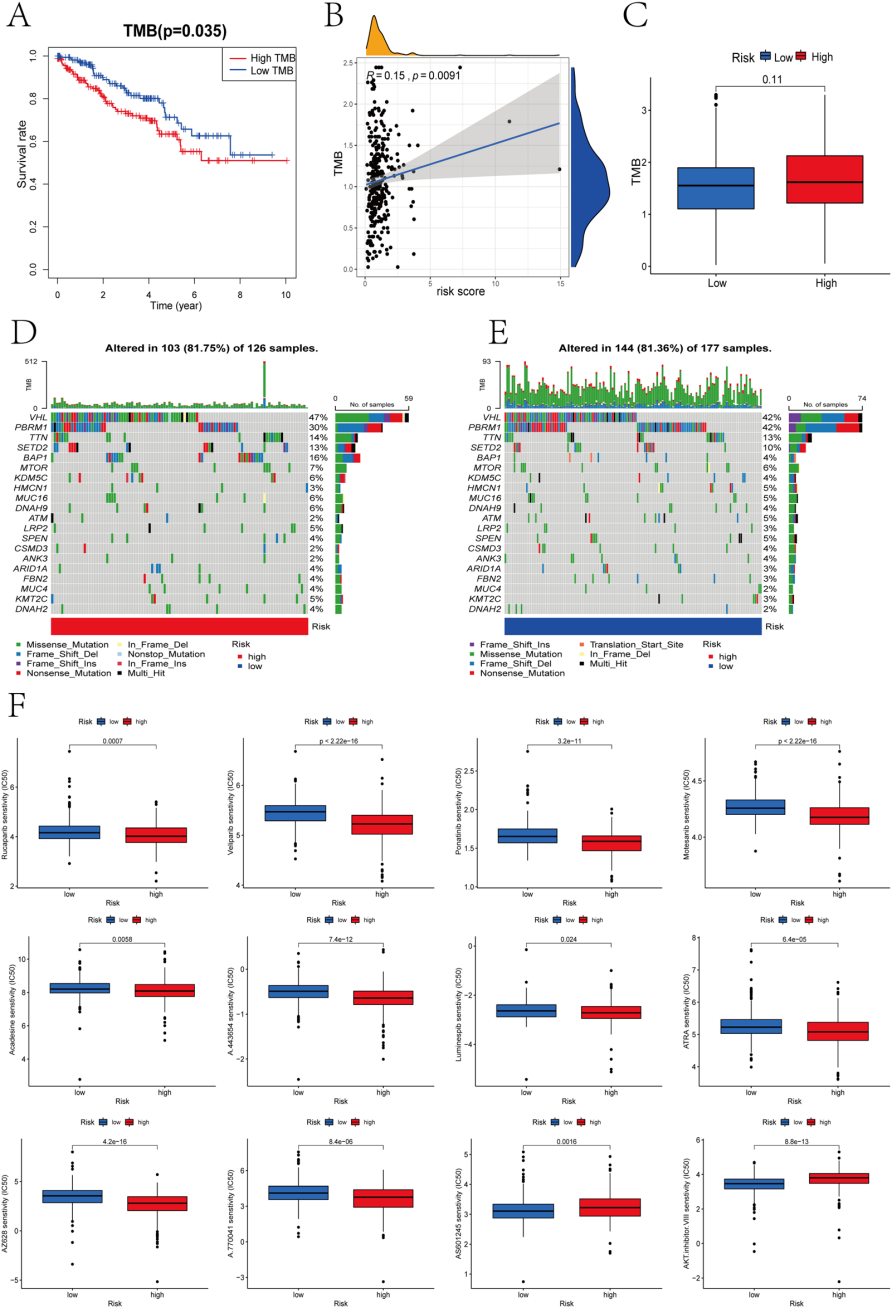
Mutation and drug sensitivity analysis. (**A**) Kaplan–Meier curves between low- and high-TMB groups. (**B**) Correlations between PRGP_score and TMB. (**C**) The difference of TMB in two groups. (**D**,**E**) The distribution variations of somatic mutations between two groups. (**F**) The difference of IC50 of some drugs between two groups.

Compared with low TMB group, the high TMB group had worse OS (Fig. 8A), and the PRGP_score was positively related to TMB (Fig. 8B). Interestingly, statistical difference in TMB between high- and low-risk group was no observed (Fig. 8C). Then, we further analyzed the distribution variations of somatic mutations between two groups in TCGA-KIRC cohort. Missense mutation is the most usual type of mutation, followed by frame shift deletions and nonsense mutation. The largest differences in mutations between two groups were PBRM1 and BAP1 mutations. Specifically, BAP1 mutations were more common in high-risk group (16% vs.4%), while PBRM1 mutations were more common in low-risk group (42% vs.30%) (Fig. 8D,E). Further exploration found that both PBRM1 and BAP1 mutations affect the response and prognosis of ccRCC patients to immunotherapy^27^. Furthermore, we also assessed the susceptibility of two groups to some drugs via the R package "pRRophetic". The IC50 of these drugs was strongly different between two groups (Fig. 8F), such as Veliparib, rucaparib, Acadesine and Ponatini.

### Development of a nomogram

**Figure 9:**
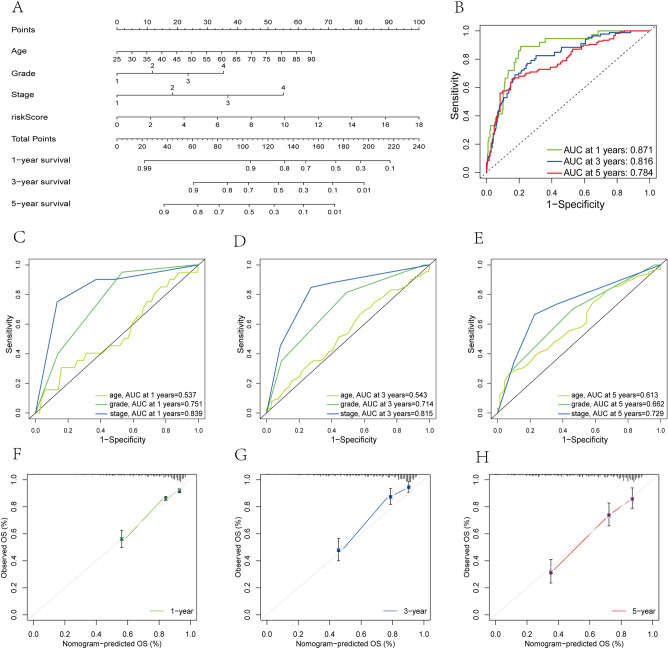
Development of a nomogram for predicting survival. (**A**) The Nomogram for predicting the OS of 1-, 3-, and 5-year of ccRCC patients. (**B**) The ROC curves of the Nomogram for predicting the survival rates of 1-, 3-, and 5-year. (**C**–**E**) The ROC curves of clinical features for predicting the survival rates of 1-, 3-, and 5-year. (**F**–H) Calibration curves of the nomogram for predicting survival rates of 1-, 3-, and 5-year.

In the TCGA entire cohort, univariate and multivariate Cox regression analysis demonstrated that age, grade, and AJCC were independent prognostic factors for ccRCC. Considering the importance of these clinical features, we built a nomogram combining the PRGP_score and these clinical parameters to predict patients' OS (Fig. 9A). The AUC values of the nomogram to predict OS of 1-, 3-, and 5-year were 0.871, 0.816 and 0.784, respectively (Fig. 9B), which were better than those of any single clinicopathological parameter (Fig. 9C–E), showing a better survival prediction ability. The calibration plots also revealed the stable performance of the nomogram (Fig. 9F-H), which is helpful for clinical application.

## Discussion

Pyroptosis is a novel programmed and inflammatory death discovered following apoptosis and necrosis^16^. Studies have found that pyroptosis is strongly associated with various diseases^28–31^, especially malignant tumors^32^. However, the conclusions about the correlation between pyroptosis and tumors are not completely consistent. Awad et al. found that NLRP1 can regulate the caspase-1-dependant secretion of pro-inflammatory interleukin (IL)1β and IL18 cytokines, thereby promoting the development of skin cancer^33^. This suggests that inflammatory factors released by pyroptosis can form a microenvironment suitable for tumor cell growth and cancer progression. Conversely, Gao et al. found that down-regulation of the pyroptotic gene GSDMD inhibited tumor proliferation through the intrinsic mitochondrial apoptosis pathway and inhibition of EGFR/Akt signaling pathway^34^. It suggests that pyroptosis may play a dual role in the pathogenesis of tumors, and also shows the heterogeneity of tumors and the complexity of the immune microenvironment.

In the study, we aim to explore the expression pattern and genetic variation of PRGs in ccRCC as well as their effect on TME and prognostic value. Compared with normal renal tissue, 32 PRGs were up-regulated and 9 PRGs were down-regulated in ccRCC tissue. Prognostic analysis showed that 21 PRGs were related to the prognosis of ccRCC, indicating that PRGs may play a vital role in the occurrence and progression of ccRCC. In addition, PRGs in ccRCC have low somatic mutations and CNV, showing a stable genetic pattern. We run consensus clustering analysis on patients in TCGA-KIRC cohort based on 41 differentially expressed PRGs and obtained three pyroptosis-related subtypes, namely C1, C2, and C3. Obvious differences in PRG expression, OS, PFS, clinical features, and tumor immune microenvironment infiltration were observed among three subtypes. The PRGs of the subtype C2 are highly expressed with the worst prognosis and are strongly associated with worse grade, AJCC stage, and TNM stage. Functional enrichment analysis and GSEA analysis were performed to investigate the functional differences in different subtypes. We identified some immune-related biological processes and cancer-related pathways, such as T cell activation, proliferation and regulation, lymphocyte activation and proliferation regulation, T cell receptor signaling pathway, NOD-like receptor signaling pathway, PD-L1 expression and PD-1 checkpoint pathway in cancer, and NF-kappa B signaling pathway. Past studies have demonstrated that the proliferation and activation of some immune cells can affect the microenvironment and lead to tumor progression. Togashi et al. found that Tregs in TME can inhibit anti-tumor immunity through various mechanisms including affecting the production of CTLA-4 and immunosuppressive cytokines TGF-βand IL-10^35^. Li et al. found that B cells recruited into kidney tumors may participate in tumor migration and metastasis via IL-1β/HIF-2 α signaling^36^. Similarly, some related signaling pathways also play key role in tumorigenesis and progression. Liu et al. found that NOD-like receptors (NLRs) have been regarded as crucial regulators in inflammation-associated tumorigenesis, angiogenesis, cancer cell stemness, and chemoresistance^37^. These evidence suggests that these biological processes and cancer-related pathways we identified based on pyroptosis-related subtypes are of great significance, which may be the important reasons for differences in prognostic and clinical features among three subtypes. Tumor and immune metabolism and immune cell metabolism are another important components of TME, which can regulate anti-tumor immunity and affect the response to immunotherapy^38^. Regards as a metabolically driven disease, ccRCC had the characteristic of mutations in target genes involved in metabolic pathways^39^. Several metabolomic studies have revealed different processes covered by its metabolic reprogramming, including aerobic glycolysis, fatty acid metabolism, and utilization of tryptophan, glutamine, and arginine^39–43^. These findings are largely consistent with our study. Metabolic-related pathways were strongly enriched in subtype C3 according to the results of GSVA, such as insulin signaling pathway, amino acid degradation, TCA cycle, and adipocytokine signaling pathway. Combining with previous studies^44^, we speculated that the metabolism and function of immune cells in subtype C3 microenvironment are more active. While immune cells in subtype C2 microenvironment may have metabolic dysfunction and glucose utilization disorder, thus enriching immune deficiency in GSVA analysis. Since there are few studies on pyroptosis in ccRCC, we speculated that pyroptosis may affect the occurrence and progression of tumors by affecting the immune-related metabolism and immune cell metabolism of TME. However, the specific mechanisms and their regulation may need to be explored in further experimental research.

Patients with ccRCC are insensitive to traditional chemotherapy and radiotherapy. Despite recent promising advances in immunotherapy, the prognosis of patients is heterogeneous and the response to immunotherapy is unsatisfactory, revealing the crucial impact of TME in tumorigenesis and progression of ccRCC. The TME consists of cellular components, extracellular matrix (ECM), and interstitial fluid. The cellular components of TME include tumor cells, stromal cells (such as fibroblasts), endothelial cells of blood vessels and lymphatic vessels, neurons, and infiltrating immune cells^45^. Among them, immune cells are the main cellular components of TME. They can drive or prevent tumor progression by participating in various immune responses and activities^46^. Increasing evidence shows the central role of TME in tumorigenesis, immune escape, progression, and metastasis^47^. In this study, compared with the other two subtypes, we found that subtype C2 had a higher level of immune cell infiltration, such as T cells CD8, T cells gamma delta, and NK cells activated. And these cells are considered to be effector cells that can kill tumor cells^48–50^. The ESTIMATE algorithm showed that subtype C2 had the highest immune score and matrix score, suggesting its higher content of immune cells and stromal cells. However, the subtype C2 has the worst prognosis, indicating the complexity of the TME in ccRCC. Recent studies have found that in more advanced and metastatic diseases, CD 8 tumor-infiltrating lymphocytes (TIL) in ccRCC transform to terminal depletion phenotype, express multiple immune checkpoint molecules, and the diversity of T cell receptor (TCR) is limited^51^. Further researches have demonstrated that poor prognosis was strongly related to depleted polyclonal CD8 T cells expressing immune checkpoints such as PD-1, TIM-3, and LAG-3 and displaying decreased cytotoxic functionality^52,53^. Prinz et al. found that kidney tumor cells may induce NK cell dysfunction via various mechanisms including the diacylglycerol kinase, mitogen-activated protein kinase (MAPK/WEK), and TGF-β/SMAD signaling pathways^54^. These reasons may make it difficult for T cells CD8, T cells gamma delta, and NK cells to exert their anti-tumor effect in TME. In addition, Daniel Chen et al. believe that although some tumor tissues with more immune cells, these immune cells cannot penetrate into the inner core of tumor cells and are confined to the peripheral matrix of tumor cells, which is an immunophenotype known as immune-exclude tumor^55^ and is very similar to subtype C2. We speculate that the activation of the matrix in the microenvironment will lead to immunosuppression and prevent the related effector cells from killing the tumor. The effects of these comprehensive factors eventually result in a poor prognosis of subtype C2. We were surprised to find that high-risk group had a similar TME landscape to those with subtype C2 after a similar analysis in two groups based on the risk prognostic score model. Compared to low-risk group, high-risk group had a higher infiltration levels of T cells CD8 and NK cells activated, and the highest immune and stromal scores, but like the subtype C2 with the worst prognosis. Furthermore, we noted that both the subtype C2 and high-risk group of patients had a higher infiltration level of Tregs and T cells follicular helper. We already know that Tregs in TME can inhibit anti-tumor immunity via multiple mechanisms, which is related to poor prognosis. Based on this evidence, we speculate that pyroptosis can affect immune cells infiltration in TME and usually leads to immunosuppression. This suggests that targeting pyroptosis may reverse the immunosuppression of the microenvironment and enhance the efficacy of immunotherapy.

ICIs targeting PD-1/PD-L1 has become the mainstay treatments of many cancer. Now many additional immune checkpoints have also become the main targets of research, including CTLA-4, PD-L2, LAG-3, TIM-3, and so on^56^. High expression levels of immune checkpoints such as PD-1 were found in both the subtype C2 and the high-risk group. Generally, the higher the level of gene expression in immune checkpoints, the higher its response to ICIs. However, the opposite result was found in our study. Patients with subtype C2 and high risk groups had a higher TIDE scores, suggesting that they had less benefit from ICIs. Based on previous study^57^, we speculate that pyroptosis may recruit functionally restricted immune cells, thus weakening the efficacy of ICIs and promoting the progress of ccRCC. This again suggests that targeting pyroptosis may be a new direction for improving the efficacy of immunotherapy. In addition, we also assessed the susceptibility of high- and low-risk group to some anticancer drugs. According to IC50, high-risk group patients may be more sensitive to Ponatini, Veliparib, rucaparib and Acadesine, while the low-risk group may be more sensitive to AS601245 and AKT inhibitor. As the third generation kinase inhibitor, Ponatinib is an effective drug for hematological malignancies^58^. Garner et al. found that Ponatinib can inhibit polyclonal drug-resistant KIT oncoproteins and reveals therapeutic potential in heavily pretreated gastrointestinal stromal tumor (GIST) patients^59^. Pletcher et al. found that Veliparib and rucaparib can induce apoptosis of RCC cell and reduce tumor cell growth and proliferation^60^. Woodard et al. found that Acadesine could inhibit the growth and promote apoptosis of RCC cells via inducing AMPK activity and inhibiting mTOR and its effectors^61^. Recently, Liang et al. found that Acadesine combined with rapamycin can reduce the proliferation of ccRCC cells, increase apoptosis, and significantly reduce the expression of p-Akt, HIF-2 α and vascular endothelial growth factor in mouse kidney tumor tissue^62^. There are many similar studies^63,64^. Therefore, the drugs we found may have potential therapeutic effects on ccRCC.

In the present study, we also constructed a risk prognosis score mode. The PRGP_score based on 4 genes can accurately predict the prognosis of ccRCC patients and effectively divide patients into high- and low-risk group. Compared to low-risk group, high-risk group had a poor OS and was related to worse T stage, M stage, grade, and AJCC stage. We verified the stability and effectiveness of the model in testing cohort , TCGA entire cohort and E-MTAB-1980 external cohort. In addition, stratified analyses showed that PRGP_score was applicable in different clinical subgroups. Univariate and multivariate Cox prognostic analysis demonstrated that the PRGP_score was an independent prognostic factor for ccRCC. Subsequently, integrating PRGP_score with age, grade, and AJCC stage, we constructed a nomogram to further improve the practical application of PRGP_score in clinic.

We have to admit that there are some deficiencies in our research. all analyses used the data from public databases, requiring prospective and larger trials to provide high-level evidence for clinical application. Then, our research suggests that PRGs have potential roles in the TME, clinicopathological features and prognosis of ccRCC, but the specific mechanisms of their effects and how they are regulated need to be further studied.

## Conclusion

We systematically analyzed the genetic variation and expression profiles of PRGs in ccRCC. Based on the differentially expressed PRGs, we identified three pyroptosis-related subtypes, and developed and verified a risk model to predict the prognosis of patients. Remarkable differences in clinical features and TME among different subtypes were observed. Our study demonstrates that PRGs may play a crucial role in the TME, clinicopathological features and prognosis of ccRCC, which provides a new idea for development of effective immunotherapy strategies.

### Supplementary Information

Supplementary Information 1.

Supplementary Figures.

## Data Availability

The data and information analyzed for this study can be obtained from the TCGA database (https://portal.gdc.cancer.gov/repository, TCGA-KIRC), ArrayExpress database (https://www.ebi.ac.uk/biostudies/arrayexpress/, E-MTAB-1980), MSigDB database (http://www.broad.mit.edu/gsea/msigdb/, REACTOME_PYROPTOSIS), Xena Functional Genomics Explorer (https://xenabrowser.net/datapages/, KIRC), and the TIDE website (http://tide.dfci.harvard.edu/). The TIDE data are available in the supplementary data zip files S1. The datasets analysed during the current study are also available from the corresponding author on reasonable request.

## Abbreviations

ccRCC: Clear cell renal cell carcinoma
PRGs: Pyroptosis-related genes
TCGA: The cancer genome atlas
TME: Tumor microenvironment
DEGs: Differentially expressed genes
OS: Overall survival
PFS: Progression free survival
ICIs: Immune checkpoint inhibitors
RCC: Renal cell carcinoma
TIIC: Tumor infiltrating immune cells
FPKM: Fragments per kilobase million
STRING: The search tool for the retrieval of interacting genes
PPI: Protein–protein interaction
CNV: Copy number variation
PCA: Principal component analysis
GSVA: Gene set variation analysis
GO: Gene ontology
KEGG: Kyoto encyclopedia of genes and genomes
TMB: Tumor mutation burden
TIDE: The tumor immune dysfunction and exclusion

## References

[1] Sung H, Ferlay J, Siegel RL (2021). Global cancer statistics 2020: GLOBOCAN estimates of incidence and mortality worldwide for 36 cancers in 185 countries. CA Cancer J. Clin..

[2] Bray F, Ferlay J, Soerjomataram I, Siegel RL, Torre LA, Jemal A (2018). Global cancer statistics 2018: GLOBOCAN estimates of incidence and mortality worldwide for 36 cancers in 185 countries. CA Cancer J. Clin..

[3] Siegel RL, Miller KD, Jemal A (2019). Cancer statistics, 2019. CA Cancer J. Clin..

[4] Chen VJ, Hernandez-Meza G, Agrawal P (2019). Time on therapy for at least three months correlates with overall survival in metastatic renal cell carcinoma. Cancers (Basel).

[5] Bedke J, Stuhler V, Stenzl A, Brehmer B (2018). Immunotherapy for kidney cancer: Status quo and the future. Curr. Opin. Urol..

[6] Barata PC, Rini BI (2017). Treatment of renal cell carcinoma: Current status and future directions. CA Cancer J. Clin..

[7] Considine B, Hurwitz ME (2019). Current status and future directions of immunotherapy in renal cell carcinoma. Curr. Oncol. Rep..

[8] Senbabaoglu Y, Gejman RS, Winer AG (2016). Tumor immune microenvironment characterization in clear cell renal cell carcinoma identifies prognostic and immunotherapeutically relevant messenger RNA signatures. Genome Biol..

[9] Atkins MB, Tannir NM (2018). Current and emerging therapies for first-line treatment of metastatic clear cell renal cell carcinoma. Cancer Treat. Rev..

[10] Diaz-Montero CM, Rini BI, Finke JH (2020). The immunology of renal cell carcinoma. Nat. Rev. Nephrol..

[11] Fang Y, Tian S, Pan Y (2020). Pyroptosis: A new frontier in cancer. Biomed. Pharmacother..

[12] Ruan J, Wang S, Wang J (2020). Mechanism and regulation of pyroptosis-mediated in cancer cell death. Chem. Biol. Interact..

[13] Shi J, Gao W, Shao F (2017). Pyroptosis: Gasdermin-mediated programmed necrotic cell death. Trends Biochem. Sci..

[14] He WT, Wan H, Hu L (2015). Gasdermin D is an executor of pyroptosis and required for interleukin-1beta secretion. Cell Res..

[15] Al MA, Mimi AA, Aziz MA (2021). Role of pyroptosis in cancer and its therapeutic regulation. Eur. J. Pharmacol..

[16] Xia X, Wang X, Cheng Z (2019). The role of pyroptosis in cancer: Pro-cancer or pro-"host"?. Cell Death Dis..

[17] Karki R, Kanneganti TD (2019). Diverging inflammasome signals in tumorigenesis and potential targeting. Nat. Rev. Cancer.

[18] Erkes DA, Cai W, Sanchez IM (2020). Mutant BRAF and MEK inhibitors regulate the tumor immune microenvironment via pyroptosis. Cancer Discov..

[19] Runa F, Hamalian S, Meade K, Shisgal P, Gray PC, Kelber JA (2017). Tumor microenvironment heterogeneity: Challenges and opportunities. Curr. Mol. Biol. Rep..

[20] Iyengar NM, Gucalp A, Dannenberg AJ, Hudis CA (2016). Obesity and cancer mechanisms: Tumor microenvironment and inflammation. J. Clin. Oncol..

[21] Binnewies M, Roberts EW, Kersten K (2018). Understanding the tumor immune microenvironment (TIME) for effective therapy. Nat. Med..

[22] Noessner E, Brech D, Mendler AN, Masouris I, Schlenker R, Prinz PU (2012). Intratumoral alterations of dendritic-cell differentiation and CD8(+) T-cell anergy are immune escape mechanisms of clear cell renal cell carcinoma. Oncoimmunology.

[23] Lee CH, Motzer RJ (2016). Immune checkpoint therapy in renal cell carcinoma. Cancer J..

[24] Zhang C, Duan Y, Xia M (2019). TFEB mediates immune evasion and resistance to mTOR inhibition of renal cell carcinoma via induction of PD-L1. Clin. Cancer Res..

[25] Lee SS, Cheah YK (2019). The interplay between MicroRNAs and cellular components of tumour microenvironment (TME) on non-small-cell lung cancer (NSCLC) progression. J. Immunol. Res..

[26] Song W, Ren J, Xiang R, Kong C, Fu T (2021). Identification of pyroptosis-related subtypes, the development of a prognosis model, and characterization of tumor microenvironment infiltration in colorectal cancer. Oncoimmunology.

[27] Aili A, Wen J, Xue L, Wang J (2021). Mutational analysis of PBRM1 and significance of PBRM1 mutation in Anti-PD-1 immunotherapy of clear cell renal cell carcinoma. Front. Oncol..

[28] He B, Nie Q, Wang F (2021). Role of pyroptosis in atherosclerosis and its therapeutic implications. J. Cell Physiol..

[29] Lei Q, Yi T, Chen C (2018). NF-kappaB-Gasdermin d (GSDMD) axis couples oxidative stress and NACHT, LRR and PYD Domains-Containing protein 3 (NLRP3) Inflammasome-Mediated cardiomyocyte pyroptosis following myocardial infarction. Med. Sci. Monit..

[30] Mckenzie BA, Mamik MK, Saito LB (2018). Caspase-1 inhibition prevents glial inflammasome activation and pyroptosis in models of multiple sclerosis. Proc. Natl. Acad. Sci. U S A.

[31] Al MA, Mimi AA, Zaeem M (2021). Role of pyroptosis in diabetic retinopathy and its therapeutic implications. Eur. J. Pharmacol..

[32] Tan Y, Chen Q, Li X (2021). Pyroptosis: A new paradigm of cell death for fighting against cancer. J. Exp. Clin. Cancer Res..

[33] Awad F, Assrawi E, Louvrier C (2018). Photoaging and skin cancer: Is the inflammasome the missing link?. Mech. Ageing Dev..

[34] Gao J, Qiu X, Xi G (2018). Downregulation of GSDMD attenuates tumor proliferation via the intrinsic mitochondrial apoptotic pathway and inhibition of EGFR/Akt signaling and predicts a good prognosis in nonsmall cell lung cancer. Oncol. Rep..

[35] Togashi Y, Shitara K, Nishikawa H (2019). Regulatory T cells in cancer immunosuppression—implications for anticancer therapy. Nat. Rev. Clin. Oncol..

[36] Li S, Huang C, Hu G (2020). Tumor-educated B cells promote renal cancer metastasis via inducing the IL-1beta/HIF-2alpha/Notch1 signals. Cell Death Dis..

[37] Liu P, Lu Z, Liu L (2019). NOD-like receptor signaling in inflammation-associated cancers: From functions to targeted therapies. Phytomedicine.

[38] Leone RD, Powell JD (2020). Metabolism of immune cells in cancer. Nat. Rev. Cancer.

[39] Lucarelli G, Loizzo D, Franzin R (2019). Metabolomic insights into pathophysiological mechanisms and biomarker discovery in clear cell renal cell carcinoma. Expert Rev. Mol. Diagn..

[40] Ragone R, Sallustio F, Piccinonna S (2016). Renal cell carcinoma: A study through NMR-Based metabolomics combined with transcriptomics. Diseases.

[41] Lucarelli G, Rutigliano M, Sallustio F (2018). Integrated multi-omics characterization reveals a distinctive metabolic signature and the role of NDUFA4L2 in promoting angiogenesis, chemoresistance, and mitochondrial dysfunction in clear cell renal cell carcinoma. Aging (Albany NY).

[42] Bombelli S, Torsello B, De Marco S (2020). 36-KDa annexin a3 isoform negatively modulates lipid storage in clear cell renal cell carcinoma cells. Am. J. Pathol..

[43] Bianchi C, Meregalli C, Bombelli S (2017). The glucose and lipid metabolism reprogramming is grade-dependent in clear cell renal cell carcinoma primary cultures and is targetable to modulate cell viability and proliferation. Oncotarget.

[44] Beckermann K, Siska P, Mason F, Rathmell K, Rathmell JC (2017). Metabolic barriers to immunotherapy in renal cell carcinoma. J. Clin. Oncol..

[45] Kozlova N, Grossman JE, Iwanicki MP, Muranen T (2020). The interplay of the extracellular matrix and stromal cells as a drug target in Stroma-Rich cancers. Trends Pharmacol. Sci..

[46] Seager RJ, Hajal C, Spill F, Kamm RD, Zaman MH (2017). Dynamic interplay between tumour, stroma and immune system can drive or prevent tumour progression. Converg. Sci. Phys. Oncol..

[47] Hinshaw DC, Shevde LA (2019). The tumor microenvironment innately modulates cancer progression. Cancer Res..

[48] Farhood B, Najafi M, Mortezaee K (2019). CD8(+) cytotoxic T lymphocytes in cancer immunotherapy: A review. J. Cell Physiol..

[49] Ma R, Yuan D, Guo Y, Yan R, Li K (2020). Immune effects of gammadelta t cells in colorectal cancer: A review. Front. Immunol..

[50] Eckl J, Buchner A, Prinz PU (2012). Transcript signature predicts tissue NK cell content and defines renal cell carcinoma subgroups independent of TNM staging. J. Mol. Med. (Berl).

[51] Braun DA, Street K, Burke KP (2021). Progressive immune dysfunction with advancing disease stage in renal cell carcinoma. Cancer Cell.

[52] Giraldo NA, Becht E, Pages F (2015). Orchestration and prognostic significance of immune checkpoints in the microenvironment of primary and metastatic renal cell cancer. Clin. Cancer Res..

[53] Giraldo NA, Becht E, Vano Y (2017). Tumor-Infiltrating and peripheral blood t-cell immunophenotypes predict early relapse in localized clear cell renal cell carcinoma. Clin. Cancer Res..

[54] Prinz PU, Mendler AN, Brech D, Masouris I, Oberneder R, Noessner E (2014). NK-cell dysfunction in human renal carcinoma reveals diacylglycerol kinase as key regulator and target for therapeutic intervention. Int. J. Cancer.

[55] Chen DS, Mellman I (2017). Elements of cancer immunity and the cancer-immune set point. Nature.

[56] Liao G, Wang P, Wang Y (2021). Identification of the prognosis value and potential mechanism of immune checkpoints in renal clear cell carcinoma microenvironment. Front. Oncol..

[57] Zhang Z, Zhang Y, Xia S (2020). Gasdermin E suppresses tumour growth by activating anti-tumour immunity. Nature.

[58] Massaro F, Molica M, Breccia M (2018). Ponatinib: A review of efficacy and safety. Curr. Cancer Drug Targets.

[59] Garner AP, Gozgit JM, Anjum R (2014). Ponatinib inhibits polyclonal drug-resistant KIT oncoproteins and shows therapeutic potential in heavily pretreated gastrointestinal stromal tumor (GIST) patients. Clin. Cancer Res..

[60] Pletcher JP, Bhattacharjee S, Doan JP (2021). The emerging role of poly (ADP-Ribose) polymerase inhibitors as effective therapeutic agents in renal cell carcinoma. Front. Oncol..

[61] Woodard J, Joshi S, Viollet B, Hay N, Platanias LC (2010). AMPK as a therapeutic target in renal cell carcinoma. Cancer Biol. Ther..

[62] Liang S, Medina EA, Li B, Habib SL (2018). Preclinical evidence of the enhanced effectiveness of combined rapamycin and AICAR in reducing kidney cancer. Mol. Oncol..

[63] Wu Q, Wu W, Jacevic V, Franca T, Wang X, Kuca K (2020). Selective inhibitors for JNK signalling: A potential targeted therapy in cancer. J. Enzyme Inhib. Med. Chem..

[64] Revathidevi S, Munirajan AK (2019). Akt in cancer: Mediator and more. Semin. Cancer Biol..

