# Supplementary material for: Identification of pyroptosis-related subtypes and comprehensive analysis of characteristics of the tumor microenvironment infiltration in clear cell renal cell carcinoma
Source: Sci Rep. 2023 Sep 25;13:16055. doi: 10.1038/s41598-023-43023-y (PMC10519968; doi:10.1038/s41598-023-43023-y)
Supplement: Supplementary file 1 — Supplementary Information 1. [file 41598_2023_43023_MOESM1_ESM.zip › Approval Letter ofthe Research Ethics Committee.pdf]

# 广东省人民医院（广东省医学科学院）
# 医学研究伦理委员会
Research Ethics Committee
Guangdong Provincial People's Hospital, Guangdong Academy of Medical Sciences


粤医科伦理 2019413H（R1）号
No.GDREC2019413H(R1)

## 医学研究伦理委员会审查批件
## Approval Letter of the Research Ethics Committee

| 项目名称<br>**Study Title** | PD-1/PD-L1 信号通过 CD8+CD28- T 细胞参与肾移植外周免疫耐受的机制研究 | | | |
|---|---|---|---|---|
| 项目负责人<br>**Principal investigator** | 余玉明 | 项目类型及编号<br>**Project No. and Issued BY** | 广东省自然科学基金 | |
| 审查文件<br>**Reviewed items** | 研究方案版本号<br>**Protocol No.** | 1.0 | 研究方案日期<br>**Protocol dated** | 2019.10.28 |
| | 知情同意书版本号<br>**Informed consent form No.** | 1.0（患者）<br>2.0（志愿者） | 知情同意书日期<br>**Informed consent form dated** | 2019.10.28（患者）<br>20200518（志愿者） |
| | 其他文件<br>**Other specify** | 授权职责表等。 | | |
| 审查类型<br>**Types of review** | 修后快审 | 委员会人数<br>**Number of reviewers** | 13 | |
| 评审结果<br>**Evaluation results** | ● 批准（Approved）<br>○ 修改后批准（Approved after revision）*<br>○ 修改后再审（Re-reviewed after revision）*<br>○ 不批准（No approval）*<br>○ 暂停或终止研究（Suspension or Termination of the approved research）*<br>* 评审意见另附（Comments in the attachment） | | | |

主任委员
**Signature Chair:**__________ 评审日期 **Date of review:** 2020 年 07 月 16 日

（印章：广东省人民医院 医学研究伦理委员会）

注：1. 为充分保证研究对象的权益，本项试验开始后如有研究方案、知情同意书和招募材料等改变，或出现严重不良事件（人体）或其它意外情况，请速报本委员会，本委员会拥有是否终止该试验的权利。
2. 研究期限超过一年的项目应将年度进展报告提交伦理委员会跟踪审查。
3. 研究结束后请将研究课题结题报告提交伦理委员会审查。

本委员会依据《赫尔辛基宣言》等伦理原则和国家法规的要求操作。

# 广东省人民医院（广东省医学科学院）
# 医学研究伦理委员会文件

Research Ethics Committee

Guangdong Provincial People's Hospital, Guangdong Academy of Medical Sciences

粤医科伦理 2019413A 号

**No.GDREC**2019413A

## 医学研究伦理委员会审查批件

**Approval Letter of the Research Ethics Committee**

<table>
<tr><td>项目名称<br>**Study Title**</td><td colspan="4">PD-1/PD-L1 信号通过 CD8+CD28- T 细胞参与肾移植外周免疫耐受的机制研究</td></tr>
<tr><td>项目负责人<br>**Principal investigator**</td><td>余玉明</td><td>项目类型及编号<br>**Approval NO. and Issued BY**</td><td colspan="2">广东省自然科学基金</td></tr>
<tr><td rowspan="2">审查文件<br>**Reviewed items**</td><td>研究方案版本号<br>**Protocol No.**</td><td>1.0</td><td>研究方案日期<br>**Protocol dated**</td><td>2019.10.28</td></tr>
<tr><td>其他文件<br>**Other specify**</td><td colspan="3">动物伦理审查申请附表等。</td></tr>
<tr><td>动物品种品系<br>**The species or strains of animals**</td><td>NOG 小鼠（重症免疫缺陷小鼠）</td><td>动物数量<br>**Number of animals**</td><td colspan="2">18 只</td></tr>
<tr><td>评审方式<br>**Reviewed methods**</td><td>快速审查</td><td>委员会人数<br>**No. of reviewers**</td><td colspan="2">13</td></tr>
<tr><td>评审结果<br>**Evaluation results**</td><td colspan="4">● **批准（Approved）***<br>○ 修改后批准（Approved after revision）*<br>○ 修正后再审（Re-reviewed after revision）*<br>○ 不批准（No approval）*<br>○ 暂停或终止研究（Suspension or Termination of the approved research）*<br>* 评审意见另附（Comments in the attachment）</td></tr>
<tr><td colspan="5">主任委员<br>**Signature of Chair:** [signature]<br>评审日期<br>**Date of review:** 2019 年 12 月 31 日<br>[Stamp: 广东省人民医院 医学研究伦理委员会]</td></tr>
<tr><td colspan="5">注：本委员会依据国家科技部《关于善待实验动物的指导性意见》和相应的国际动物实验伦理原则(*Guide for the Care and Use of Laboratory Animals*)的要求操作，请研究者在实验中遵循有关准则。</td></tr>
</table>
