# Supplementary material for: Identification of pyroptosis-related subtypes and comprehensive analysis of characteristics of the tumor microenvironment infiltration in clear cell renal cell carcinoma
Source: Sci Rep. 2023 Sep 25;13:16055. doi: 10.1038/s41598-023-43023-y (PMC10519968; doi:10.1038/s41598-023-43023-y)
Supplement: Supplementary file 1 — Supplementary Information 1. [file 41598_2023_43023_MOESM1_ESM.zip › Clinical information of 491 KIRC patients.pdf]

Clinical information of 491 KIRC patients

Id	futime	fustat	age	gender	grade	stage	T	M	N
TCGA-BP-4165	3037	Alive	64	FEMALE	G1	Stage I	T1b	M0	N0
TCGA-B4-5836	141	Alive	61	FEMALE	G2	Stage I	T1b	M0	N0
TCGA-B4-5378	175	Alive	62	MALE	G2	Stage I	T1	M0	N0
TCGA-B8-4154	255	Alive	73	FEMALE	G2	Stage I	T1a	M0	N0
TCGA-B2-5633	358	Alive	56	MALE	G2	Stage I	T1b	M0	N0
TCGA-BP-4756	374	Alive	62	FEMALE	G2	Stage I	T1b	M0	N0
TCGA-3Z-A93Z	385	Alive	69	MALE	G2	Stage I	T1a	M0	N0
TCGA-BP-4768	400	Alive	72	FEMALE	G2	Stage I	T1a	M0	N0
TCGA-B8-5553	435	Alive	67	FEMALE	G2	Stage I	T1b	M0	N0
TCGA-B0-5120	493	Alive	72	FEMALE	G2	Stage I	T1a	M0	N0
TCGA-B8-5546	505	Alive	38	FEMALE	G2	Stage I	T1b	M0	N0
TCGA-B8-5545	522	Alive	42	MALE	G2	Stage I	T1a	M0	N0
TCGA-B0-5121	554	Alive	56	MALE	G2	Stage I	T1b	M0	N0
TCGA-A3-3380	567	Alive	54	MALE	G2	Stage I	T1	M0	N0
TCGA-A3-3387	617	Alive	49	MALE	G2	Stage I	T1a	M0	N0
TCGA-BP-4795	620	Alive	74	FEMALE	G2	Stage I	T1a	M0	N0
TCGA-B0-5399	652	Alive	46	MALE	G2	Stage I	T1b	M0	N0
TCGA-CZ-5988	693	Alive	38	MALE	G2	Stage I	T1b	M0	N0
TCGA-BP-5186	693	Alive	50	FEMALE	G2	Stage I	T1a	M0	N0
TCGA-BP-5006	840	Alive	61	MALE	G2	Stage I	T1a	M0	N0
TCGA-B0-5700	1082	Alive	77	MALE	G2	Stage I	T1a	M0	N0
TCGA-B0-5110	1092	Alive	71	FEMALE	G2	Stage I	T1a	M0	N0
TCGA-A3-3331	1257	Alive	86	FEMALE	G2	Stage I	T1	M0	N0
TCGA-A3-3358	1307	Alive	57	FEMALE	G2	Stage I	T1a	M0	N0
TCGA-A3-3374	1314	Alive	51	FEMALE	G2	Stage I	T1b	M0	N0
TCGA-A3-3328	1385	Alive	79	MALE	G2	Stage I	T1b	M0	N0
TCGA-A3-3349	1385	Alive	34	FEMALE	G2	Stage I	T1b	M0	N0
TCGA-B0-5695	1420	Alive	61	FEMALE	G2	Stage I	T1b	M0	N0
TCGA-B0-5710	1459	Alive	57	MALE	G2	Stage I	T1b	M0	N0
TCGA-AK-3450	1508	Alive	85	FEMALE	G2	Stage I	T1a	M0	N0
TCGA-CJ-4892	1521	Alive	65	FEMALE	G2	Stage I	T1b	M0	N0
TCGA-A3-3362	1559	Alive	60	FEMALE	G2	Stage I	T1a	M0	N0
TCGA-B0-5702	1605	Alive	71	MALE	G2	Stage I	T1b	M0	N0
TCGA-A3-3329	1624	Alive	75	MALE	G2	Stage I	T1b	M0	N0
TCGA-CZ-4859	1787	Alive	59	FEMALE	G2	Stage I	T1	M0	N0
TCGA-B0-5697	1835	Alive	50	MALE	G2	Stage I	T1a	M0	N0
TCGA-BP-4964	1862	Alive	54	FEMALE	G2	Stage I	T1a	M0	N0
TCGA-A3-3385	1993	Alive	46	FEMALE	G2	Stage I	T1a	M0	N0
TCGA-A3-3370	2274	Alive	48	FEMALE	G2	Stage I	T1b	M0	N0
TCGA-A3-3359	2504	Alive	82	FEMALE	G2	Stage I	T1a	M0	N0
TCGA-B0-5699	2741	Alive	53	MALE	G2	Stage I	T1	M0	N0
TCGA-BP-4325	2964	Alive	64	FEMALE	G2	Stage I	T1b	M0	N0
TCGA-BP-4162	3074	Alive	65	FEMALE	G2	Stage I	T1b	M0	N0
TCGA-CW-6088	3222	Alive	60	MALE	G2	Stage I	T1b	M0	N0
TCGA-AK-3425	3343	Alive	68	MALE	G2	Stage I	T1	M0	N0
TCGA-BP-4158	3377	Alive	69	MALE	G2	Stage I	T1b	M0	N0
TCGA-B0-5705	3668	Alive	65	FEMALE	G2	Stage I	T1	M0	N0
TCGA-B8-5549	194	Alive	53	MALE	G3	Stage I	T1b	M0	N0
TCGA-B8-5159	240	Alive	61	FEMALE	G3	Stage I	T1a	M0	N0
TCGA-B2-5641	324	Alive	79	MALE	G3	Stage I	T1a	M0	N0
TCGA-CZ-5986	373	Alive	61	MALE	G3	Stage I	T1	M0	N0
TCGA-B8-4148	379	Alive	63	FEMALE	G3	Stage I	T1a	M0	N0

TCGA-B8-4621	431	Alive	63	MALE	G3	Stage I	T1b	M0	N0
TCGA-A3-3378	630	Alive	60	MALE	G3	Stage I	T1	M0	N0
TCGA-BP-4986	785	Alive	75	MALE	G3	Stage I	T1a	M0	N0
TCGA-A3-3306	1120	Alive	67	MALE	G3	Stage I	T1b	M0	N0
TCGA-BP-5182	1165	Alive	56	MALE	G3	Stage I	T1a	M0	N0
TCGA-B0-5703	1203	Alive	73	MALE	G3	Stage I	T1b	M0	N0
TCGA-BP-4995	1371	Alive	68	MALE	G3	Stage I	T1b	M0	N0
TCGA-CZ-5984	1491	Alive	51	MALE	G3	Stage I	T1b	M0	N0
TCGA-A3-3373	1621	Alive	54	FEMALE	G3	Stage I	T1b	M0	N0
TCGA-BP-4968	1746	Alive	40	MALE	G3	Stage I	T1b	M0	N0
TCGA-A3-3367	2270	Alive	72	MALE	G3	Stage I	T1b	M0	N0
TCGA-CJ-4874	2283	Alive	73	FEMALE	G3	Stage I	T1b	M0	N0
TCGA-B0-5698	2583	Alive	77	MALE	G3	Stage I	T1b	M0	N0
TCGA-B0-5707	2828	Alive	39	FEMALE	G3	Stage I	T1a	M0	N0
TCGA-BP-4338	2859	Alive	43	MALE	G3	Stage I	T1b	M0	N0
TCGA-B0-5691	3431	Alive	66	FEMALE	G3	Stage I	T1a	M0	N0
TCGA-BP-5169	193	Alive	70	MALE	G4	Stage I	T1b	M0	N0
TCGA-CJ-4872	1435	Alive	51	MALE	G4	Stage I	T1b	M0	N0
TCGA-AK-3427	2566	Alive	65	MALE	GX	Stage I	T1a	M0	N0
TCGA-A3-3363	319	Alive	50	MALE	G2	Stage II	T2	M0	N0
TCGA-A3-3351	910	Alive	42	MALE	G2	Stage II	T2a	M0	N0
TCGA-BP-5007	1140	Alive	45	MALE	G2	Stage II	T2	M0	N0
TCGA-A3-3317	1491	Alive	67	MALE	G2	Stage II	T2	M0	N0
TCGA-CZ-5452	1556	Alive	69	MALE	G2	Stage II	T2	M0	N0
TCGA-CZ-5989	1599	Alive	60	MALE	G2	Stage II	T2	M0	N0
TCGA-CZ-5985	1629	Alive	58	MALE	G2	Stage II	T2	M0	N0
TCGA-AK-3429	2017	Alive	54	FEMALE	G2	Stage II	T2	M0	N0
TCGA-CJ-4639	2308	Alive	49	FEMALE	G2	Stage II	T2	M0	N0
TCGA-B0-5706	2414	Alive	45	MALE	G2	Stage II	T2	M0	N0
TCGA-A3-3343	945	Alive	79	MALE	G3	Stage II	T2	M0	N0
TCGA-AK-3456	1143	Alive	48	MALE	G3	Stage II	T2	M0	N0
TCGA-AK-3451	1481	Alive	48	MALE	G3	Stage II	T2	M0	N0
TCGA-CZ-5456	1558	Alive	57	MALE	G3	Stage II	T2	M0	N0
TCGA-CZ-5451	1668	Alive	74	MALE	G3	Stage II	T2	M0	N0
TCGA-CJ-4643	1793	Alive	67	FEMALE	G3	Stage II	T2b	M0	N0
TCGA-BP-4174	1879	Alive	49	MALE	G3	Stage II	T2	M0	N0
TCGA-BP-4173	1893	Alive	47	MALE	G3	Stage II	T2	M0	N0
TCGA-CJ-4876	1955	Alive	57	MALE	G3	Stage II	T2b	M0	N0
TCGA-BP-4960	2172	Alive	46	MALE	G3	Stage II	T2	M0	N0
TCGA-A3-3357	2688	Alive	62	MALE	G3	Stage II	T2	M0	N0
TCGA-BP-5199	1355	Alive	58	MALE	G4	Stage II	T2	M0	N0
TCGA-A3-3335	1886	Alive	41	MALE	G4	Stage II	T2a	M0	N0
TCGA-AK-3443	1423	Alive	45	MALE	GX	Stage II	T2	M0	N0
TCGA-AK-3433	2192	Alive	48	FEMALE	GX	Stage II	T2	M0	N0
TCGA-B4-5832	155	Alive	65	MALE	G2	Stage III	T3b	M0	N0
TCGA-BP-4967	205	Alive	76	MALE	G2	Stage III	T3a	M0	N0
TCGA-B8-4620	226	Alive	70	FEMALE	G2	Stage III	T3a	M0	N0
TCGA-B8-4151	280	Alive	51	FEMALE	G2	Stage III	T3a	M0	N0
TCGA-B0-5113	359	Alive	69	FEMALE	G2	Stage III	T3a	M0	N0
TCGA-B0-5097	665	Alive	59	FEMALE	G2	Stage III	T3b	M0	N0
TCGA-B0-5108	911	Alive	54	MALE	G2	Stage III	T3a	M0	N0
TCGA-BP-5191	967	Alive	79	MALE	G2	Stage III	T3a	M0	N0
TCGA-BP-4351	970	Alive	51	FEMALE	G2	Stage III	T3a	M0	N0
TCGA-BP-4332	1133	Alive	36	MALE	G2	Stage III	T3a	M0	N0
TCGA-BP-4330	1888	Alive	60	FEMALE	G2	Stage III	T3a	M0	N0

TCGA-AK-3428	2223	Alive	62	MALE	G2	Stage III	T3b	M0	N0
TCGA-CW-5587	2226	Alive	62	FEMALE	G2	Stage III	T3b	M0	N0
TCGA-BP-4160	2881	Alive	67	MALE	G2	Stage III	T3a	M0	N0
TCGA-B0-4710	96	Alive	75	FEMALE	G3	Stage III	T3a	M0	N0
TCGA-BP-4989	118	Alive	58	MALE	G3	Stage III	T3a	M0	N0
TCGA-B8-5550	434	Alive	71	MALE	G3	Stage III	T3a	M0	N0
TCGA-BP-5198	603	Alive	72	MALE	G3	Stage III	T3b	M0	N0
TCGA-B0-5116	657	Alive	52	MALE	G3	Stage III	T3b	M0	N0
TCGA-BP-4797	1107	Alive	34	MALE	G3	Stage III	T3b	M0	N0
TCGA-A3-3307	1436	Alive	66	MALE	G3	Stage III	T3b	M0	N0
TCGA-B0-5692	1487	Alive	66	FEMALE	G3	Stage III	T3b	M0	N0
TCGA-BP-4971	1487	Alive	40	MALE	G3	Stage III	T3a	M0	N0
TCGA-BP-4345	1516	Alive	62	MALE	G3	Stage III	T3b	M0	N0
TCGA-CZ-4863	1691	Alive	51	FEMALE	G3	Stage III	T3b	M0	N0
TCGA-CJ-4873	1776	Alive	85	FEMALE	G3	Stage III	T3a	M0	N0
TCGA-B0-5713	1865	Alive	75	FEMALE	G3	Stage III	T3b	M0	N0
TCGA-CJ-4636	1924	Alive	51	MALE	G3	Stage III	T3a	M0	N0
TCGA-BP-4163	2839	Alive	60	FEMALE	G3	Stage III	T3a	M0	N0
TCGA-B0-5400	1132	Alive	59	FEMALE	G4	Stage III	T3b	M0	N0
TCGA-B0-5696	1727	Alive	69	MALE	G4	Stage III	T3a	M0	N0
TCGA-B0-5701	1732	Alive	65	MALE	G4	Stage III	T3b	M0	N0
TCGA-CJ-4640	1998	Alive	49	MALE	G4	Stage III	T3a	M0	N0
TCGA-AK-3436	2044	Alive	40	MALE	G2	Stage IV	T2	M1	N0
TCGA-CW-5591	2271	Alive	56	MALE	G2	Stage IV	T3a	M1	N0
TCGA-CW-5585	2609	Alive	51	MALE	G2	Stage IV	T3b	M1	N0
TCGA-B8-4622	181	Alive	57	MALE	G3	Stage IV	T3a	M1	N0
TCGA-B4-5377	365	Alive	68	FEMALE	G3	Stage IV	T3	M1	N0
TCGA-B0-5115	797	Alive	43	MALE	G3	Stage IV	T2	M1	N0
TCGA-CJ-4904	1792	Alive	60	FEMALE	G3	Stage IV	T3a	M1	N0
TCGA-B0-5712	2722	Alive	68	FEMALE	G3	Stage IV	T2	M1	N0
TCGA-BP-5201	951	Alive	63	MALE	G4	Stage IV	T3b	M1	N0
TCGA-CJ-4890	2085	Alive	72	MALE	G4	Stage IV	T3a	M1	N0
TCGA-G6-A8L7	2133	Alive	81	FEMALE	G3	Stage I	T1b	MX	N0
TCGA-B8-A54H	256	Alive	69	FEMALE	G3	Stage II	T2a	MX	N0
TCGA-CJ-4869	2554	Alive	49	MALE	G2	Stage III	T2	M0	N1
TCGA-BP-4970	433	Alive	44	MALE	G3	Stage III	T1a	M0	N1
TCGA-BP-4761	182	Alive	57	MALE	G4	Stage III	T3a	M0	N1
TCGA-B8-5158	293	Alive	56	MALE	G4	Stage III	T3a	M0	N1
TCGA-EU-5904	551	Alive	47	FEMALE	G1	Stage I	T1	M0	NX
TCGA-A3-3323	1106	Alive	53	MALE	G1	Stage I	T1b	M0	NX
TCGA-A3-3326	1137	Alive	47	MALE	G1	Stage I	T1a	M0	NX
TCGA-A3-3320	1508	Alive	52	FEMALE	G1	Stage I	T1b	M0	NX
TCGA-B0-5690	2408	Alive	53	FEMALE	G1	Stage I	T1b	M0	NX
TCGA-CW-6093	3146	Alive	73	MALE	G1	Stage I	T1a	M0	NX
TCGA-B2-4102	202	Alive	61	MALE	G2	Stage I	T1b	M0	NX
TCGA-EU-5906	206	Alive	55	MALE	G2	Stage I	T1b	M0	NX
TCGA-B2-5636	265	Alive	79	MALE	G2	Stage I	T1a	M0	NX
TCGA-B2-5635	315	Alive	74	MALE	G2	Stage I	T1a	M0	NX
TCGA-BP-4782	354	Alive	55	FEMALE	G2	Stage I	T1a	M0	NX
TCGA-DV-5569	355	Alive	29	FEMALE	G2	Stage I	T1a	M0	NX
TCGA-DV-5568	370	Alive	26	MALE	G2	Stage I	T1a	M0	NX
TCGA-B2-3924	371	Alive	73	MALE	G2	Stage I	T1b	M0	NX
TCGA-BP-4349	372	Alive	68	FEMALE	G2	Stage I	T1a	M0	NX
TCGA-B8-5552	392	Alive	41	FEMALE	G2	Stage I	T1b	M0	NX
TCGA-BP-5187	406	Alive	54	MALE	G2	Stage I	T1a	M0	NX

TCGA-BP-5194	408	Alive	39	MALE	G2	Stage I	T1a	M0	NX
TCGA-BP-4776	411	Alive	52	MALE	G2	Stage I	T1a	M0	NX
TCGA-B8-4146	511	Alive	41	FEMALE	G2	Stage I	T1b	M0	NX
TCGA-B0-5117	535	Alive	40	MALE	G2	Stage I	T1b	M0	NX
TCGA-BP-5192	714	Alive	59	MALE	G2	Stage I	T1a	M0	NX
TCGA-DV-5574	723	Alive	37	MALE	G2	Stage I	T1a	M0	NX
TCGA-BP-5195	749	Alive	75	MALE	G2	Stage I	T1a	M0	NX
TCGA-CZ-4853	774	Alive	82	MALE	G2	Stage I	T1a	M0	NX
TCGA-AK-3461	853	Alive	72	MALE	G2	Stage I	T1a	M0	NX
TCGA-A3-3383	861	Alive	52	MALE	G2	Stage I	T1	M0	NX
TCGA-A3-3365	873	Alive	46	MALE	G2	Stage I	T1a	M0	NX
TCGA-DV-5567	910	Alive	40	FEMALE	G2	Stage I	T1a	M0	NX
TCGA-AK-3460	951	Alive	58	MALE	G2	Stage I	T1a	M0	NX
TCGA-DV-5575	1006	Alive	52	FEMALE	G2	Stage I	T1a	M0	NX
TCGA-BP-5196	1018	Alive	53	MALE	G2	Stage I	T1a	M0	NX
TCGA-BP-5008	1071	Alive	46	MALE	G2	Stage I	T1a	M0	NX
TCGA-BP-4987	1124	Alive	41	FEMALE	G2	Stage I	T1b	M0	NX
TCGA-BP-4801	1124	Alive	57	MALE	G2	Stage I	T1a	M0	NX
TCGA-A3-3319	1130	Alive	70	MALE	G2	Stage I	T1b	M0	NX
TCGA-DV-5573	1130	Alive	41	MALE	G2	Stage I	T1a	M0	NX
TCGA-BP-5001	1177	Alive	43	FEMALE	G2	Stage I	T1b	M0	NX
TCGA-BP-4999	1266	Alive	56	MALE	G2	Stage I	T1a	M0	NX
TCGA-DV-5565	1329	Alive	59	MALE	G2	Stage I	T1a	M0	NX
TCGA-DV-5566	1398	Alive	67	FEMALE	G2	Stage I	T1a	M0	NX
TCGA-BP-4991	1413	Alive	54	MALE	G2	Stage I	T1a	M0	NX
TCGA-BP-4804	1459	Alive	59	MALE	G2	Stage I	T1b	M0	NX
TCGA-AK-3444	1471	Alive	80	FEMALE	G2	Stage I	T1b	M0	NX
TCGA-A3-3322	1478	Alive	51	MALE	G2	Stage I	T1a	M0	NX
TCGA-BP-4789	1489	Alive	48	MALE	G2	Stage I	T1a	M0	NX
TCGA-BP-5181	1495	Alive	58	FEMALE	G2	Stage I	T1b	M0	NX
TCGA-CJ-4905	1496	Alive	62	FEMALE	G2	Stage I	T1a	M0	NX
TCGA-CJ-4899	1528	Alive	42	MALE	G2	Stage I	T1b	M0	NX
TCGA-CJ-4908	1531	Alive	38	MALE	G2	Stage I	T1a	M0	NX
TCGA-BP-4344	1666	Alive	75	FEMALE	G2	Stage I	T1a	M0	NX
TCGA-BP-4177	1670	Alive	65	MALE	G2	Stage I	T1a	M0	NX
TCGA-BP-4969	1794	Alive	63	FEMALE	G2	Stage I	T1a	M0	NX
TCGA-CJ-4634	1820	Alive	60	FEMALE	G2	Stage I	T1b	M0	NX
TCGA-BP-4775	1843	Alive	55	FEMALE	G2	Stage I	T1a	M0	NX
TCGA-CZ-4862	1843	Alive	46	MALE	G2	Stage I	T1b	M0	NX
TCGA-BP-4784	1854	Alive	67	FEMALE	G2	Stage I	T1a	M0	NX
TCGA-BP-4965	1871	Alive	46	MALE	G2	Stage I	T1a	M0	NX
TCGA-BP-4769	1876	Alive	63	MALE	G2	Stage I	T1a	M0	NX
TCGA-BP-4774	1885	Alive	57	FEMALE	G2	Stage I	T1a	M0	NX
TCGA-BP-4961	1935	Alive	47	MALE	G2	Stage I	T1a	M0	NX
TCGA-BP-4176	1955	Alive	64	MALE	G2	Stage I	T1b	M0	NX
TCGA-CW-5588	2017	Alive	78	FEMALE	G2	Stage I	T1a	M0	NX
TCGA-CZ-5982	2042	Alive	59	FEMALE	G2	Stage I	T1a	M0	NX
TCGA-AK-3434	2087	Alive	72	MALE	G2	Stage I	T1b	M0	NX
TCGA-BP-4765	2184	Alive	43	MALE	G2	Stage I	T1a	M0	NX
TCGA-BP-4758	2208	Alive	40	MALE	G2	Stage I	T1a	M0	NX
TCGA-BP-5174	2257	Alive	45	FEMALE	G2	Stage I	T1a	M0	NX
TCGA-BP-5180	2263	Alive	53	MALE	G2	Stage I	T1a	M0	NX
TCGA-BP-4760	2361	Alive	69	MALE	G2	Stage I	T1a	M0	NX
TCGA-BP-4759	2372	Alive	50	MALE	G2	Stage I	T1a	M0	NX
TCGA-CW-5589	2378	Alive	52	MALE	G2	Stage I	T1a	M0	NX

TCGA-BP-5170	2412	Alive	55	MALE	G2	Stage I	T1a	M0	NX
TCGA-CW-5583	2489	Alive	51	FEMALE	G2	Stage I	T1a	M0	NX
TCGA-CW-6096	2701	Alive	44	FEMALE	G2	Stage I	T1a	M0	NX
TCGA-B0-5693	3076	Alive	47	FEMALE	G2	Stage I	T1b	M0	NX
TCGA-EU-5905	119	Alive	67	FEMALE	G3	Stage I	T1	M0	NX
TCGA-BP-4993	177	Alive	58	MALE	G3	Stage I	T1a	M0	NX
TCGA-BP-4807	211	Alive	42	MALE	G3	Stage I	T1a	M0	NX
TCGA-BP-5177	293	Alive	46	FEMALE	G3	Stage I	T1a	M0	NX
TCGA-B2-4099	374	Alive	83	MALE	G3	Stage I	T1a	M0	NX
TCGA-BP-4977	454	Alive	57	MALE	G3	Stage I	T1b	M0	NX
TCGA-BP-5000	563	Alive	40	MALE	G3	Stage I	T1b	M0	NX
TCGA-A3-3382	574	Alive	69	MALE	G3	Stage I	T1b	M0	NX
TCGA-CJ-4893	750	Alive	76	FEMALE	G3	Stage I	T1b	M0	NX
TCGA-AK-3454	874	Alive	84	MALE	G3	Stage I	T1b	M0	NX
TCGA-BP-5175	932	Alive	60	MALE	G3	Stage I	T1a	M0	NX
TCGA-BP-4998	932	Alive	49	MALE	G3	Stage I	T1a	M0	NX
TCGA-BP-5190	1011	Alive	61	MALE	G3	Stage I	T1a	M0	NX
TCGA-BP-4982	1014	Alive	42	MALE	G3	Stage I	T1b	M0	NX
TCGA-BP-5004	1126	Alive	53	MALE	G3	Stage I	T1a	M0	NX
TCGA-BP-5185	1132	Alive	56	MALE	G3	Stage I	T1a	M0	NX
TCGA-BP-5184	1133	Alive	54	MALE	G3	Stage I	T1a	M0	NX
TCGA-AK-3458	1168	Alive	48	MALE	G3	Stage I	T1b	M0	NX
TCGA-A3-3324	1186	Alive	51	MALE	G3	Stage I	T1b	M0	NX
TCGA-BP-4994	1308	Alive	54	MALE	G3	Stage I	T1a	M0	NX
TCGA-CJ-4635	1416	Alive	48	MALE	G3	Stage I	T1b	M0	NX
TCGA-BP-4975	1433	Alive	40	MALE	G3	Stage I	T1b	M0	NX
TCGA-BP-4766	1462	Alive	43	FEMALE	G3	Stage I	T1a	M0	NX
TCGA-CJ-4903	1560	Alive	50	MALE	G3	Stage I	T1b	M0	NX
TCGA-BP-4976	1632	Alive	77	MALE	G3	Stage I	T1a	M0	NX
TCGA-BP-4777	1731	Alive	46	MALE	G3	Stage I	T1a	M0	NX
TCGA-AK-3440	1745	Alive	58	MALE	G3	Stage I	T1a	M0	NX
TCGA-CZ-4866	1768	Alive	79	FEMALE	G3	Stage I	T1	M0	NX
TCGA-BP-4963	1834	Alive	63	MALE	G3	Stage I	T1b	M0	NX
TCGA-CJ-5683	1889	Alive	78	MALE	G3	Stage I	T1b	M0	NX
TCGA-CJ-6031	1906	Alive	54	MALE	G3	Stage I	T1b	M0	NX
TCGA-CJ-5671	1943	Alive	51	MALE	G3	Stage I	T1a	M0	NX
TCGA-CJ-4886	1952	Alive	42	FEMALE	G3	Stage I	T1a	M0	NX
TCGA-CJ-5686	2038	Alive	59	FEMALE	G3	Stage I	T1b	M0	NX
TCGA-BP-4781	2080	Alive	78	MALE	G3	Stage I	T1a	M0	NX
TCGA-CW-6090	2552	Alive	68	MALE	G3	Stage I	T1b	M0	NX
TCGA-BP-4959	2660	Alive	49	MALE	G3	Stage I	T1b	M0	NX
TCGA-BP-4161	2746	Alive	74	MALE	G3	Stage I	T1b	M0	NX
TCGA-CW-5581	2799	Alive	44	MALE	G3	Stage I	T1b	M0	NX
TCGA-B0-5812	2963	Alive	53	MALE	G3	Stage I	T1b	M0	NX
TCGA-BP-4992	501	Alive	66	MALE	G4	Stage I	T1b	M0	NX
TCGA-A3-3336	1043	Alive	75	FEMALE	G4	Stage I	T1	M0	NX
TCGA-CJ-6027	1855	Alive	77	MALE	G4	Stage I	T1a	M0	NX
TCGA-CJ-4889	1946	Alive	63	FEMALE	G4	Stage I	T1a	M0	NX
TCGA-AK-3465	369	Alive	71	FEMALE	GX	Stage I	T1b	M0	NX
TCGA-B2-3923	362	Alive	59	MALE	G2	Stage II	T2	M0	NX
TCGA-CZ-5463	662	Alive	76	MALE	G2	Stage II	T2	M0	NX
TCGA-AK-3447	1217	Alive	83	MALE	G2	Stage II	T2	M0	NX
TCGA-AK-3453	1397	Alive	58	FEMALE	G2	Stage II	T2	M0	NX
TCGA-CJ-4642	1628	Alive	47	MALE	G2	Stage II	T2	M0	NX
TCGA-BP-4962	1785	Alive	58	MALE	G2	Stage II	T2	M0	NX

TCGA-B2-4101	188	Alive	52	MALE	G3	Stage II	T2a	M0	NX
TCGA-A3-3316	1493	Alive	57	MALE	G3	Stage II	T2	M0	NX
TCGA-CJ-4912	1657	Alive	61	MALE	G3	Stage II	T2	M0	NX
TCGA-AK-3431	1853	Alive	62	FEMALE	G3	Stage II	T2	M0	NX
TCGA-CJ-5675	2430	Alive	70	MALE	G3	Stage II	T2a	M0	NX
TCGA-CJ-6032	2548	Alive	63	FEMALE	G3	Stage II	T2	M0	NX
TCGA-BP-5200	1063	Alive	44	MALE	G4	Stage II	T2	M0	NX
TCGA-CZ-4858	1943	Alive	39	MALE	G4	Stage II	T2	M0	NX
TCGA-B0-4718	616	Alive	57	MALE	G2	Stage III	T3a	M0	NX
TCGA-CZ-5466	685	Alive	67	MALE	G2	Stage III	T3a	M0	NX
TCGA-A3-3372	735	Alive	64	MALE	G2	Stage III	T3	M0	NX
TCGA-BP-4347	1367	Alive	74	MALE	G2	Stage III	T3b	M0	NX
TCGA-CZ-5465	1377	Alive	76	FEMALE	G2	Stage III	T3b	M0	NX
TCGA-CJ-4870	1498	Alive	58	FEMALE	G2	Stage III	T3a	M0	NX
TCGA-CJ-4878	2186	Alive	71	FEMALE	G2	Stage III	T3a	M0	NX
TCGA-CJ-5684	2231	Alive	61	MALE	G2	Stage III	T3a	M0	NX
TCGA-BP-4167	2718	Alive	59	MALE	G2	Stage III	T3a	M0	NX
TCGA-EU-5907	127	Alive	81	MALE	G3	Stage III	T3a	M0	NX
TCGA-BP-4803	204	Alive	79	MALE	G3	Stage III	T3a	M0	NX
TCGA-B8-4153	405	Alive	74	MALE	G3	Stage III	T3a	M0	NX
TCGA-AK-3445	1280	Alive	69	MALE	G3	Stage III	T3a	M0	NX
TCGA-BP-5183	1291	Alive	57	MALE	G3	Stage III	T3a	M0	NX
TCGA-CJ-4916	1373	Alive	69	FEMALE	G3	Stage III	T3a	M0	NX
TCGA-BP-4973	1384	Alive	47	MALE	G3	Stage III	T3a	M0	NX
TCGA-CJ-4901	1450	Alive	47	MALE	G3	Stage III	T3b	M0	NX
TCGA-CJ-4907	1499	Alive	58	MALE	G3	Stage III	T3b	M0	NX
TCGA-BP-4972	1502	Alive	43	FEMALE	G3	Stage III	T3a	M0	NX
TCGA-CZ-5459	1515	Alive	63	MALE	G3	Stage III	T3b	M0	NX
TCGA-CJ-4902	1520	Alive	61	MALE	G3	Stage III	T3a	M0	NX
TCGA-CZ-5458	1558	Alive	43	MALE	G3	Stage III	T3a	M0	NX
TCGA-CJ-4884	1759	Alive	72	FEMALE	G3	Stage III	T3a	M0	NX
TCGA-CJ-4897	1808	Alive	79	FEMALE	G3	Stage III	T3a	M0	NX
TCGA-CJ-4882	1883	Alive	57	MALE	G3	Stage III	T3a	M0	NX
TCGA-CJ-4881	2014	Alive	41	MALE	G3	Stage III	T3a	M0	NX
TCGA-CJ-5676	2575	Alive	47	MALE	G3	Stage III	T3b	M0	NX
TCGA-B0-5711	2931	Alive	50	MALE	G3	Stage III	T3b	M0	NX
TCGA-B0-5709	3117	Alive	62	FEMALE	G3	Stage III	T3a	M0	NX
TCGA-BP-4983	1413	Alive	67	FEMALE	G4	Stage III	T3a	M0	NX
TCGA-CZ-5457	1547	Alive	62	MALE	G4	Stage III	T3a	M0	NX
TCGA-B0-5402	449	Alive	64	MALE	G4	Stage IV	T4	M0	NX
TCGA-CZ-5460	1430	Alive	55	MALE	G2	Stage IV	T3b	M1	NX
TCGA-CZ-5464	1492	Alive	69	MALE	G2	Stage IV	T3b	M1	NX
TCGA-B2-5639	417	Alive	46	MALE	G3	Stage IV	T3	M1	NX
TCGA-CJ-4885	2125	Alive	64	MALE	G3	Stage IV	T3a	M1	NX
TCGA-CJ-4875	2353	Alive	67	MALE	G3	Stage IV	T3a	M1	NX
TCGA-CJ-5682	1883	Alive	60	MALE	G4	Stage IV	T3a	M1	NX
TCGA-CJ-4871	2423	Alive	63	MALE	G4	Stage IV	T3a	M1	NX
TCGA-T7-A92I	356	Alive	47	FEMALE	G1	Stage I	T1a	MX	NX
TCGA-A3-A6NJ	468	Alive	57	FEMALE	G1	Stage I	T1a	MX	NX
TCGA-B8-A54K	469	Alive	61	MALE	G1	Stage I	T1a	MX	NX
TCGA-A3-A8OV	340	Alive	75	MALE	G2	Stage I	T1a	MX	NX
TCGA-6D-AA2E	362	Alive	68	FEMALE	G2	Stage I	T1b	MX	NX
TCGA-DV-A4VZ	365	Alive	53	MALE	G2	Stage I	T1a	MX	NX
TCGA-MW-A4E(	498	Alive	72	FEMALE	G2	Stage I	T1a	MX	NX
TCGA-B8-A54F	519	Alive	49	FEMALE	G2	Stage I	T1a	MX	NX

TCGA-A3-A6NL	689	Alive	49	FEMALE	G2	Stage I	T1b	MX	NX
TCGA-MM-A84U	700	Alive	58	FEMALE	G2	Stage I	T1a	MX	NX
TCGA-B8-A54I	150	Alive	48	MALE	G3	Stage I	T1b	MX	NX
TCGA-B8-A54E	909	Alive	62	FEMALE	G3	Stage I	T1b	MX	NX
TCGA-A3-A6NI	1018	Alive	47	MALE	G3	Stage I	T1a	MX	NX
TCGA-DV-A4W0	2008	Alive	55	MALE	G3	Stage I	T1b	MX	NX
TCGA-B8-A54J	528	Alive	60	MALE	G2	Stage II	T2a	MX	NX
TCGA-MM-A564	607	Alive	68	MALE	G2	Stage II	T2a	MX	NX
TCGA-A3-A8OW	323	Alive	37	MALE	G2	Stage III	T3a	MX	NX
TCGA-B8-A54D	830	Alive	69	MALE	G2	Stage III	T3a	MX	NX
TCGA-BP-4353	375	Dead	61	MALE	G2	Stage I	T1	M0	N0
TCGA-B0-4823	454	Dead	88	MALE	G2	Stage I	T1a	M0	N0
TCGA-BP-4340	562	Dead	70	FEMALE	G2	Stage I	T1b	M0	N0
TCGA-BP-4988	828	Dead	72	MALE	G2	Stage I	T1a	M0	N0
TCGA-CZ-4854	1404	Dead	68	MALE	G2	Stage I	T1b	M0	N0
TCGA-B0-5106	1598	Dead	64	MALE	G2	Stage I	T1a	M0	N0
TCGA-BP-4326	1625	Dead	53	FEMALE	G2	Stage I	T1b	M0	N0
TCGA-B0-4839	1639	Dead	80	FEMALE	G2	Stage I	T1b	M0	N0
TCGA-A3-3376	1696	Dead	51	MALE	G2	Stage I	T1a	M0	N0
TCGA-B0-4945	2145	Dead	75	FEMALE	G2	Stage I	T1a	M0	N0
TCGA-BP-4170	2343	Dead	72	FEMALE	G2	Stage I	T1b	M0	N0
TCGA-B0-4833	2386	Dead	82	FEMALE	G2	Stage I	T1b	M0	N0
TCGA-BP-4331	2454	Dead	52	MALE	G2	Stage I	T1a	M0	N0
TCGA-BP-4159	2601	Dead	70	MALE	G2	Stage I	T1b	M0	N0
TCGA-B0-5088	563	Dead	53	MALE	G3	Stage I	T1b	M0	N0
TCGA-A3-3313	735	Dead	59	MALE	G3	Stage I	T1b	M0	N0
TCGA-B0-4838	834	Dead	69	FEMALE	G3	Stage I	T1b	M0	N0
TCGA-B0-5083	1045	Dead	63	MALE	G3	Stage I	T1a	M0	N0
TCGA-B0-5077	1317	Dead	77	MALE	G3	Stage I	T1a	M0	N0
TCGA-B0-4837	1378	Dead	63	MALE	G3	Stage I	T1b	M0	N0
TCGA-B0-4824	1657	Dead	49	FEMALE	G3	Stage I	T1a	M0	N0
TCGA-B0-4834	2090	Dead	49	MALE	G3	Stage I	T1a	M0	N0
TCGA-CJ-6030	2299	Dead	65	MALE	G3	Stage I	T1a	M0	N0
TCGA-BP-4327	109	Dead	75	FEMALE	G2	Stage II	T2	M0	N0
TCGA-BP-4169	701	Dead	76	FEMALE	G2	Stage II	T2	M0	N0
TCGA-CZ-5469	946	Dead	41	MALE	G2	Stage II	T2	M0	N0
TCGA-B0-4852	1121	Dead	78	FEMALE	G2	Stage II	T2	M0	N0
TCGA-CZ-4864	1315	Dead	86	MALE	G3	Stage II	T2	M0	N0
TCGA-B0-4816	1371	Dead	49	MALE	G3	Stage II	T2	M0	N0
TCGA-BP-4342	2256	Dead	79	MALE	G3	Stage II	T2	M0	N0
TCGA-B0-5081	362	Dead	79	FEMALE	G2	Stage III	T3b	M0	N0
TCGA-B0-5075	637	Dead	77	FEMALE	G2	Stage III	T3a	M0	N0
TCGA-BP-4329	845	Dead	75	MALE	G2	Stage III	T3a	M0	N0
TCGA-B0-5095	245	Dead	81	MALE	G3	Stage III	T3a	M0	N0
TCGA-B0-4843	320	Dead	57	MALE	G3	Stage III	T3a	M0	N0
TCGA-B0-5694	480	Dead	71	MALE	G3	Stage III	T3a	M0	N0
TCGA-A3-3352	561	Dead	74	MALE	G3	Stage III	T3a	M0	N0
TCGA-BP-4334	645	Dead	56	MALE	G3	Stage III	T3a	M0	N0
TCGA-B0-5085	770	Dead	76	FEMALE	G3	Stage III	T3a	M0	N0
TCGA-CJ-4894	841	Dead	58	MALE	G3	Stage III	T3a	M0	N0
TCGA-B0-4696	866	Dead	58	MALE	G3	Stage III	T3a	M0	N0
TCGA-B0-4817	1019	Dead	81	MALE	G3	Stage III	T3c	M0	N0
TCGA-BP-4799	1133	Dead	70	MALE	G3	Stage III	T3b	M0	N0
TCGA-B0-4821	1230	Dead	68	FEMALE	G3	Stage III	T3b	M0	N0
TCGA-B0-4811	1417	Dead	48	MALE	G3	Stage III	T3a	M0	N0

TCGA-BP-4346	1493	Dead	57	MALE	G3	Stage III	T3b	M0	N0
TCGA-BP-4343	1912	Dead	64	MALE	G3	Stage III	T3a	M0	N0
TCGA-CJ-4891	819	Dead	57	FEMALE	G4	Stage III	T3c	M0	N0
TCGA-BP-5010	878	Dead	63	MALE	G4	Stage III	T3a	M0	N0
TCGA-B0-4827	885	Dead	77	FEMALE	G4	Stage III	T3b	M0	N0
TCGA-BP-4985	952	Dead	72	MALE	G4	Stage III	T3a	M0	N0
TCGA-B0-4842	1724	Dead	73	FEMALE	G4	Stage III	T3a	M0	N0
TCGA-BP-4770	329	Dead	73	FEMALE	G4	Stage IV	T4	M0	N0
TCGA-B0-5094	333	Dead	62	MALE	G2	Stage IV	T3b	M1	N0
TCGA-CZ-5454	722	Dead	63	MALE	G2	Stage IV	T2	M1	N0
TCGA-B0-4846	1200	Dead	52	MALE	G2	Stage IV	T3a	M1	N0
TCGA-B0-4691	139	Dead	55	MALE	G3	Stage IV	T2	M1	N0
TCGA-B0-4814	168	Dead	58	MALE	G3	Stage IV	T4	M1	N0
TCGA-B0-4701	238	Dead	66	FEMALE	G3	Stage IV	T3a	M1	N0
TCGA-CJ-4644	336	Dead	48	FEMALE	G3	Stage IV	T3a	M1	N0
TCGA-B0-5080	342	Dead	63	MALE	G3	Stage IV	T3a	M1	N0
TCGA-B0-5092	459	Dead	53	FEMALE	G3	Stage IV	T1a	M1	N0
TCGA-BP-4335	475	Dead	65	FEMALE	G3	Stage IV	T3a	M1	N0
TCGA-CJ-5678	574	Dead	62	MALE	G3	Stage IV	T2b	M1	N0
TCGA-CJ-4868	646	Dead	42	MALE	G3	Stage IV	T3a	M1	N0
TCGA-B8-4143	709	Dead	66	FEMALE	G3	Stage IV	T3a	M1	N0
TCGA-CZ-4857	1432	Dead	56	MALE	G3	Stage IV	T3a	M1	N0
TCGA-CJ-4918	93	Dead	64	MALE	G4	Stage IV	T3a	M1	N0
TCGA-B0-4688	101	Dead	46	MALE	G4	Stage IV	T4	M1	N0
TCGA-B0-4699	110	Dead	74	MALE	G4	Stage IV	T4	M1	N0
TCGA-BP-4771	162	Dead	62	MALE	G4	Stage IV	T3a	M1	N0
TCGA-B0-4703	182	Dead	51	MALE	G4	Stage IV	T3a	M1	N0
TCGA-BP-4974	211	Dead	58	MALE	G4	Stage IV	T3a	M1	N0
TCGA-CJ-6033	224	Dead	54	FEMALE	G4	Stage IV	T3a	M1	N0
TCGA-G6-A5PC	242	Dead	54	FEMALE	G4	Stage IV	T1b	M1	N0
TCGA-BP-4352	344	Dead	74	FEMALE	G4	Stage IV	T3b	M1	N0
TCGA-BP-4787	480	Dead	59	FEMALE	G4	Stage IV	T3a	M1	N0
TCGA-B0-5107	927	Dead	65	FEMALE	G4	Stage IV	T2	M1	N0
TCGA-DV-A4VX	1626	Dead	59	MALE	G4	Stage IV	T3b	MX	N0
TCGA-A3-3347	1610	Dead	76	FEMALE	G2	Stage III	T1b	M0	N1
TCGA-CW-5584	164	Dead	74	MALE	G3	Stage III	T3b	M0	N1
TCGA-B0-4810	478	Dead	47	MALE	G3	Stage III	T3a	M0	N1
TCGA-AK-3430	480	Dead	61	MALE	G3	Stage III	T3b	M0	N1
TCGA-AK-3426	885	Dead	37	MALE	G3	Stage III	T3a	M0	N1
TCGA-B0-5109	587	Dead	69	MALE	G4	Stage III	T3b	M0	N1
TCGA-B0-5084	222	Dead	33	MALE	G3	Stage IV	T3a	M1	N1
TCGA-CJ-4638	431	Dead	46	FEMALE	G4	Stage IV	T3a	M1	N1
TCGA-BP-4354	1034	Dead	40	MALE	G4	Stage IV	T4	M1	N1
TCGA-CJ-4900	1714	Dead	69	FEMALE	G4	Stage IV	T4	M1	N1
TCGA-CJ-4920	139	Dead	64	FEMALE	G2	Stage I	T1b	M0	NX
TCGA-CZ-4865	166	Dead	70	FEMALE	G2	Stage I	T1a	M0	NX
TCGA-DV-5576	727	Dead	55	FEMALE	G2	Stage I	T1a	M0	NX
TCGA-BP-4790	1111	Dead	76	MALE	G2	Stage I	T1a	M0	NX
TCGA-A3-3325	1170	Dead	52	MALE	G2	Stage I	T1a	M0	NX
TCGA-A3-3311	1191	Dead	57	MALE	G2	Stage I	T1	M0	NX
TCGA-BP-4763	1270	Dead	79	FEMALE	G2	Stage I	T1a	M0	NX
TCGA-BP-5168	1463	Dead	75	MALE	G2	Stage I	T1a	M0	NX
TCGA-BP-5176	1590	Dead	78	FEMALE	G2	Stage I	T1a	M0	NX
TCGA-A3-3346	137	Dead	68	MALE	G3	Stage I	T1b	M0	NX
TCGA-BP-5009	1092	Dead	52	MALE	G3	Stage I	T1b	M0	NX

TCGA-BP-4762	1343	Dead	42	MALE	G3	Stage I	T1a	M0	NX
TCGA-B0-5098	1584	Dead	53	FEMALE	G3	Stage I	T1	M0	NX
TCGA-CJ-5672	1972	Dead	84	MALE	G3	Stage I	T1a	M0	NX
TCGA-B0-5102	2764	Dead	74	FEMALE	G3	Stage I	T1	M0	NX
TCGA-BP-5189	822	Dead	60	MALE	G4	Stage I	T1b	M0	NX
TCGA-CZ-4861	446	Dead	63	MALE	G2	Stage II	T2	M0	NX
TCGA-B0-4818	510	Dead	68	FEMALE	G3	Stage II	T2	M0	NX
TCGA-B0-4822	1111	Dead	78	MALE	G4	Stage II	T2	M0	NX
TCGA-B0-4713	202	Dead	76	FEMALE	G2	Stage III	T3b	M0	NX
TCGA-BP-4164	992	Dead	51	FEMALE	G2	Stage III	T3a	M0	NX
TCGA-BP-4341	1589	Dead	67	MALE	G2	Stage III	T3a	M0	NX
TCGA-B0-5099	485	Dead	88	FEMALE	G3	Stage III	T3b	M0	NX
TCGA-AK-3455	683	Dead	71	FEMALE	G3	Stage III	T3b	M0	NX
TCGA-B0-4848	883	Dead	54	MALE	G3	Stage III	T3b	M0	NX
TCGA-BP-4981	1097	Dead	75	FEMALE	G3	Stage III	T3a	M0	NX
TCGA-B0-5100	1913	Dead	72	MALE	G3	Stage III	T3a	M0	NX
TCGA-B0-4694	106	Dead	72	MALE	G4	Stage III	T3b	M0	NX
TCGA-CW-6097	571	Dead	32	MALE	G4	Stage III	T3a	M0	NX
TCGA-B0-4707	600	Dead	63	MALE	G4	Stage III	T3a	M0	NX
TCGA-CJ-5679	679	Dead	73	MALE	G4	Stage III	T3b	M0	NX
TCGA-BP-4355	953	Dead	59	FEMALE	G4	Stage III	T3a	M0	NX
TCGA-CJ-4913	1173	Dead	45	FEMALE	G4	Stage III	T3a	M0	NX
TCGA-B0-4815	1588	Dead	65	MALE	G4	Stage III	T3a	M0	NX
TCGA-CZ-5987	445	Dead	60	MALE	G2	Stage IV	T3b	M1	NX
TCGA-B0-4845	1986	Dead	70	MALE	G2	Stage IV	T3a	M1	NX
TCGA-B0-4714	99	Dead	81	MALE	G3	Stage IV	T3b	M1	NX
TCGA-B0-4841	204	Dead	63	MALE	G3	Stage IV	T2	M1	NX
TCGA-B0-4828	307	Dead	79	MALE	G3	Stage IV	T2	M1	NX
TCGA-CZ-5462	311	Dead	83	MALE	G3	Stage IV	T1b	M1	NX
TCGA-B0-4844	313	Dead	60	MALE	G3	Stage IV	T3a	M1	NX
TCGA-CJ-5681	552	Dead	44	FEMALE	G3	Stage IV	T3a	M1	NX
TCGA-B0-4847	793	Dead	60	MALE	G3	Stage IV	T3a	M1	NX
TCGA-CJ-4887	932	Dead	48	MALE	G3	Stage IV	T3a	M1	NX
TCGA-CW-5590	1075	Dead	51	MALE	G3	Stage IV	T3a	M1	NX
TCGA-B0-4836	1238	Dead	61	MALE	G3	Stage IV	T3b	M1	NX
TCGA-B0-4712	1337	Dead	76	MALE	G3	Stage IV	T3a	M1	NX
TCGA-CW-5580	1964	Dead	73	FEMALE	G3	Stage IV	T3a	M1	NX
TCGA-B0-4819	183	Dead	60	FEMALE	G4	Stage IV	T3b	M1	NX
TCGA-CZ-4860	206	Dead	60	MALE	G4	Stage IV	T4	M1	NX
TCGA-CZ-5461	330	Dead	52	MALE	G4	Stage IV	T1b	M1	NX
TCGA-CZ-5455	561	Dead	63	MALE	G4	Stage IV	T3b	M1	NX
TCGA-CJ-4923	572	Dead	63	FEMALE	G4	Stage IV	T3a	M1	NX
TCGA-B0-4697	578	Dead	46	FEMALE	G4	Stage IV	T3b	M1	NX
TCGA-CJ-5680	768	Dead	65	FEMALE	G4	Stage IV	T3a	M1	NX
TCGA-CJ-5677	782	Dead	54	FEMALE	G4	Stage IV	T3a	M1	NX
TCGA-CJ-4895	1200	Dead	62	MALE	G4	Stage IV	T3a	M1	NX
TCGA-CJ-4888	1567	Dead	59	MALE	G4	Stage IV	T3a	M1	NX
TCGA-CJ-6028	1625	Dead	58	MALE	G4	Stage IV	T3a	M1	NX
TCGA-CJ-4641	1661	Dead	55	FEMALE	G4	Stage IV	T3a	M1	NX
TCGA-BP-5178	1912	Dead	71	MALE	G4	Stage IV	T3a	M1	NX
TCGA-B0-4700	1980	Dead	60	MALE	G4	Stage IV	T4	M1	NX
TCGA-CJ-4637	2227	Dead	52	FEMALE	G4	Stage IV	T2b	M1	NX
TCGA-G6-A8L8	1091	Dead	62	FEMALE	G3	Stage I	T1b	MX	NX
TCGA-G6-A8L6	313	Dead	55	MALE	G3	Stage IV	T2a	MX	NX
