# Supplementary material for: Identification of pyroptosis-related subtypes and comprehensive analysis of characteristics of the tumor microenvironment infiltration in clear cell renal cell carcinoma
Source: Sci Rep. 2023 Sep 25;13:16055. doi: 10.1038/s41598-023-43023-y (PMC10519968; doi:10.1038/s41598-023-43023-y)
Supplement: Supplementary file 1 — Supplementary Information 1. [file 41598_2023_43023_MOESM1_ESM.zip › Patients information of the clinical transcriptome sequencing data.pdf]

Patients information of the clinical transcriptome sequencing data

Patient	Age	Gender	Laterality	Pathology	T	N	M	Stage	Grade	Sample	Type	Postion
P1	51	MALE	Left	sRCC	T4	N0	M0	Stage IV	Grade 4	P1_N1	Normal	N1
P1	51	MALE	Left	sRCC	T4	N0	M0	Stage IV	Grade 4	P1_T1	Tumor	T1
P1	51	MALE	Left	sRCC	T4	N0	M0	Stage IV	Grade 4	P1_T2	Tumor	T2
P1	51	MALE	Left	sRCC	T4	N0	M0	Stage IV	Grade 4	P1_T3	Tumor	T3
P1	51	MALE	Left	sRCC	T4	N0	M0	Stage IV	Grade 4	P1_T4	Tumor	T4
P2	71	MALE	Left	ccRCC	T1	N0	M0	Stage I	Grade 2	P2_T1	Tumor	T1
P2	71	MALE	Left	ccRCC	T1	N0	M0	Stage I	Grade 2	P2_T2	Tumor	T2
P2	71	MALE	Left	ccRCC	T1	N0	M0	Stage I	Grade 2	P2_T3	Tumor	T3
P2	71	MALE	Left	ccRCC	T1	N0	M0	Stage I	Grade 2	P2_N1	Normal	N1
P6	74	MALE	Right	ccRCC	T2	N0	M0	Stage II	Grade 1	P6_T1	Tumor	T1
P6	74	MALE	Right	ccRCC	T2	N0	M0	Stage II	Grade 1	P6_T2	Tumor	T2
P6	74	MALE	Right	ccRCC	T2	N0	M0	Stage II	Grade 1	P6_T3	Tumor	T3
P6	74	MALE	Right	ccRCC	T2	N0	M0	Stage II	Grade 1	P6_N1	Normal	N1
P7	58	MALE	Left	ccRCC	T1	N0	M0	Stage I	Grade 2	P7_T1	Tumor	T1
P7	58	MALE	Left	ccRCC	T1	N0	M0	Stage I	Grade 2	P7_T2	Tumor	T2
P7	58	MALE	Left	ccRCC	T1	N0	M0	Stage I	Grade 2	P7_T3	Tumor	T3
P7	58	MALE	Left	ccRCC	T1	N0	M0	Stage I	Grade 2	P7_N1	Normal	N1
P8	82	MALE	Right	ccRCC	T1	N1	M0	Stage III	Grade 2	P8_T2	Tumor	T2
P8	82	MALE	Right	ccRCC	T1	N1	M0	Stage III	Grade 2	P8_T1	Tumor	T1
P8	82	MALE	Right	ccRCC	T1	N1	M0	Stage III	Grade 2	P8_N1	Normal	N1
P11	70	MALE	Left	ccRCC	T1	N0	M0	Stage I	Grade 1	P11_T3	Tumor	T3
P11	70	MALE	Left	ccRCC	T1	N0	M0	Stage I	Grade 1	P11_T2	Tumor	T2
P11	70	MALE	Left	ccRCC	T1	N0	M0	Stage I	Grade 1	P11_T1	Tumor	T1
P11	70	MALE	Left	ccRCC	T1	N0	M0	Stage I	Grade 1	P11_N1	Normal	N1
