# Supplementary material for: Identification of pyroptosis-related subtypes and comprehensive analysis of characteristics of the tumor microenvironment infiltration in clear cell renal cell carcinoma
Source: Sci Rep. 2023 Sep 25;13:16055. doi: 10.1038/s41598-023-43023-y (PMC10519968; doi:10.1038/s41598-023-43023-y)
Supplement: Supplementary file 2 — Supplementary Figures. [file 41598_2023_43023_MOESM2_ESM.docx]

**Supplementary Figures**


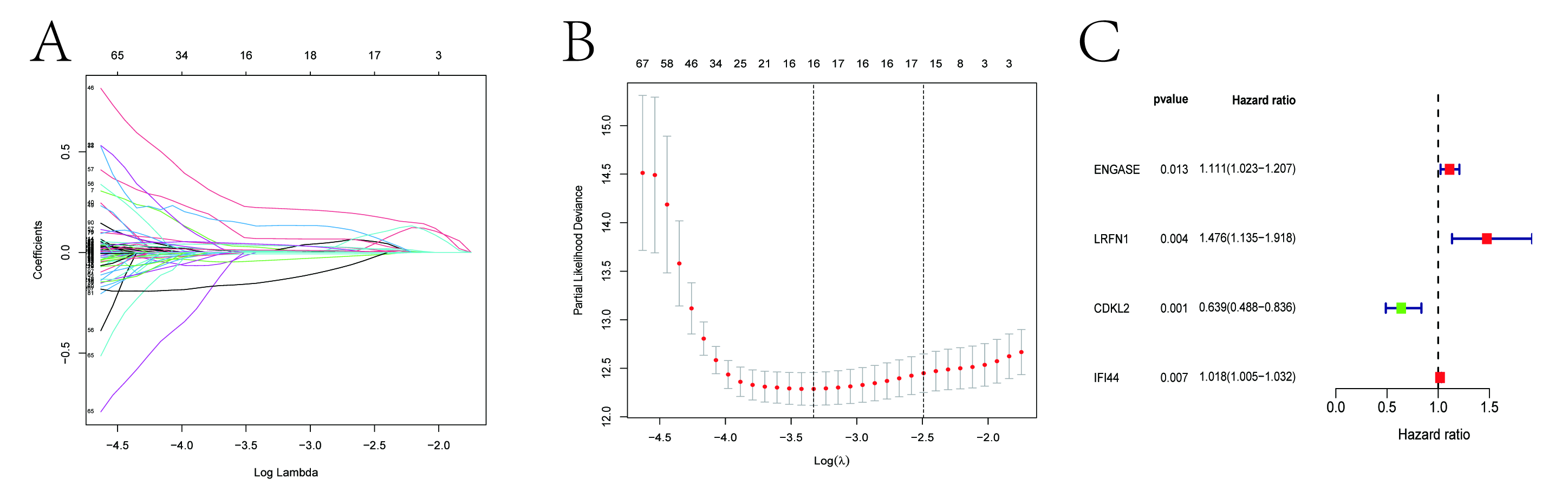


**Fig. S1.** Identifying representative prognostic genes. (A-B) The LASSO regression analysis and partial likelihood deviance on the prognostic genes. (C) Forest plot of multivariate cox regression analysis for prognostic genes.


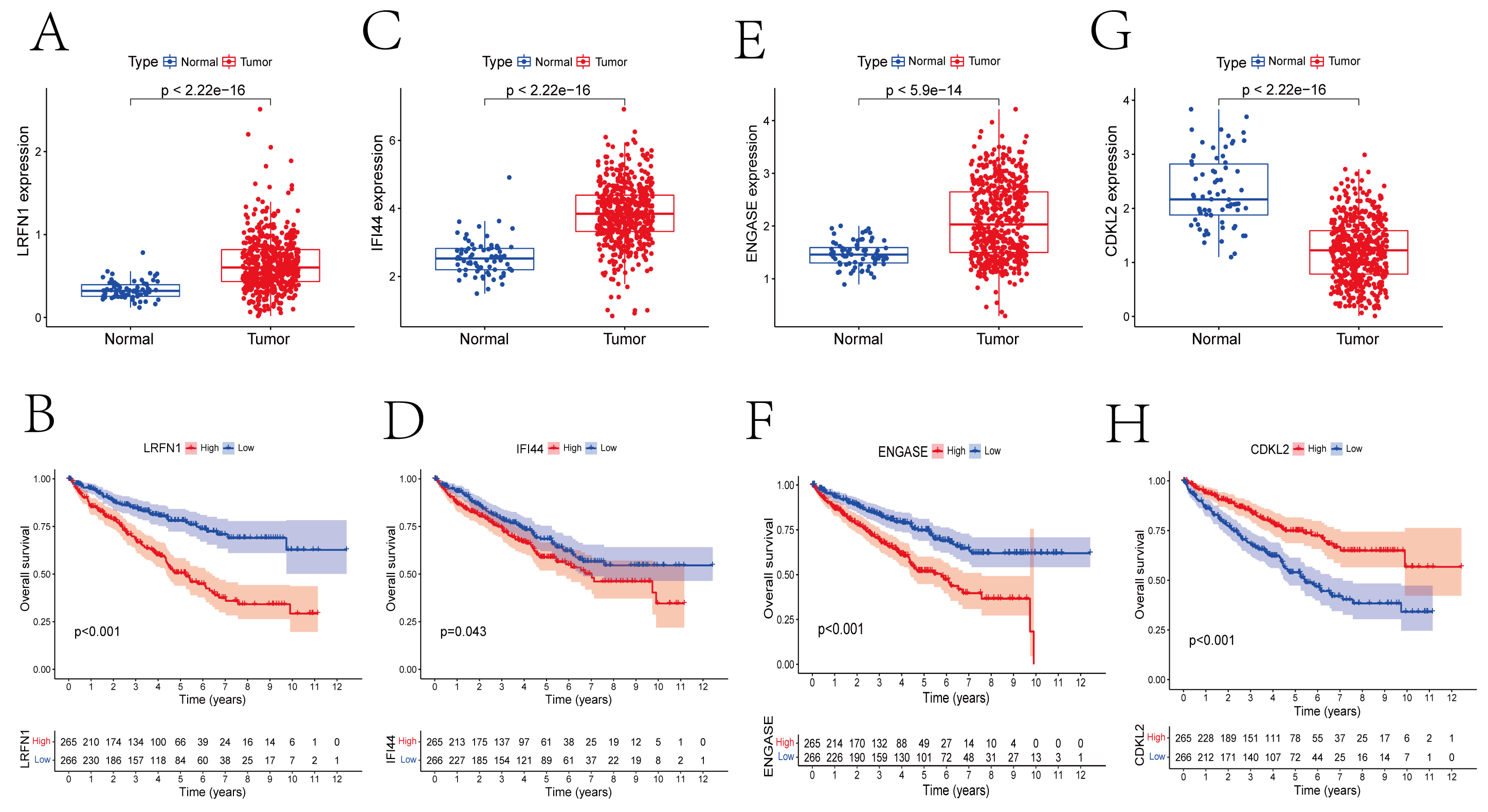


**Fig. S2.** Differential expression of 4 prognostic genes in tumor and normal tissues and their relationship with prognosis.


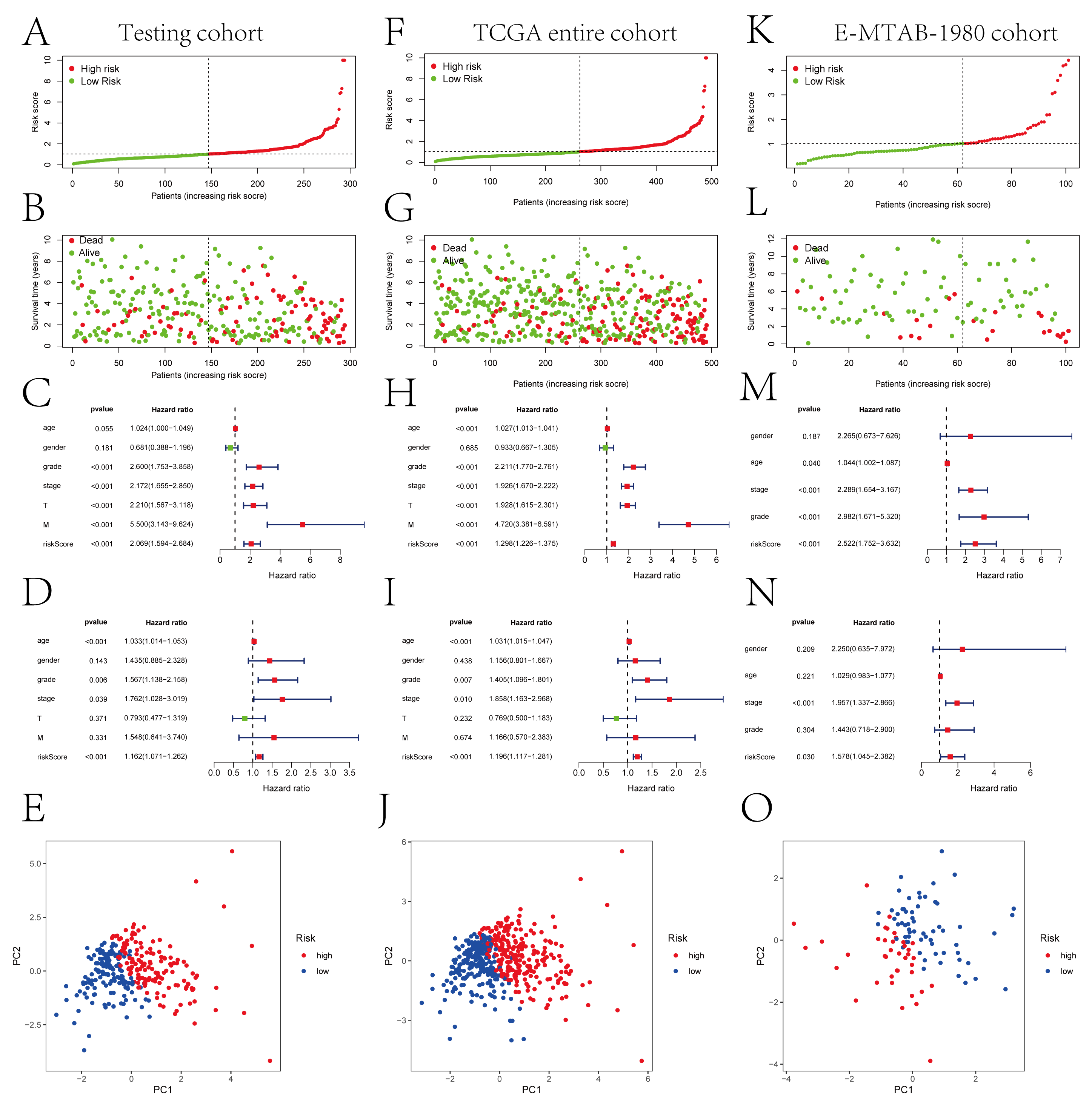


**Fig. S3.** The risk distribution curves, survival status, PCA analysis results, and univariate and multivariate Cox prognostic analysis of testing cohort (A-E), TCGA entire cohort (F-J) and E-MTAB-1980 cohort (K-O).


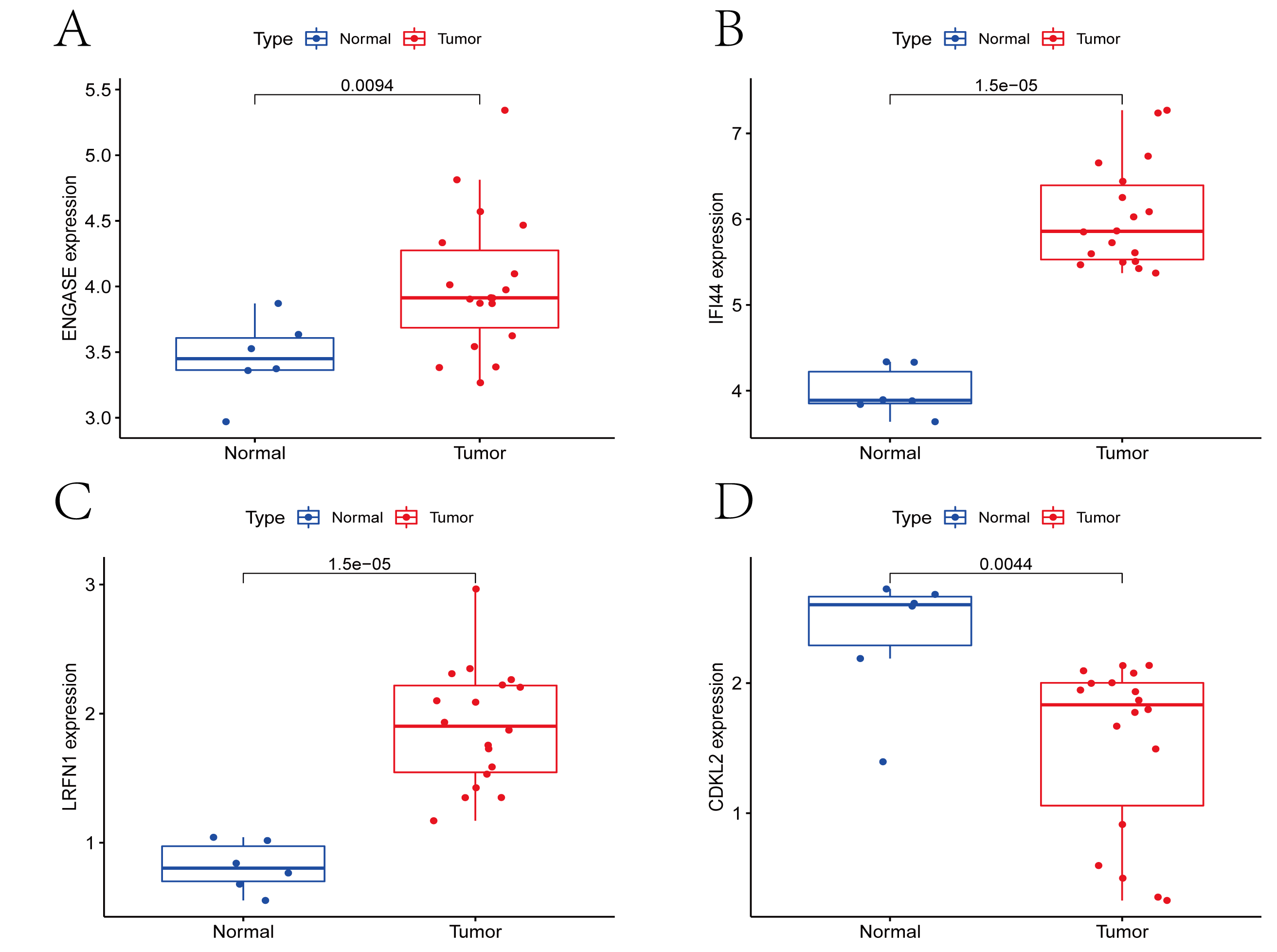


**Fig. S4.** The results of high-throughput sequencing of 4 prognostic genes.


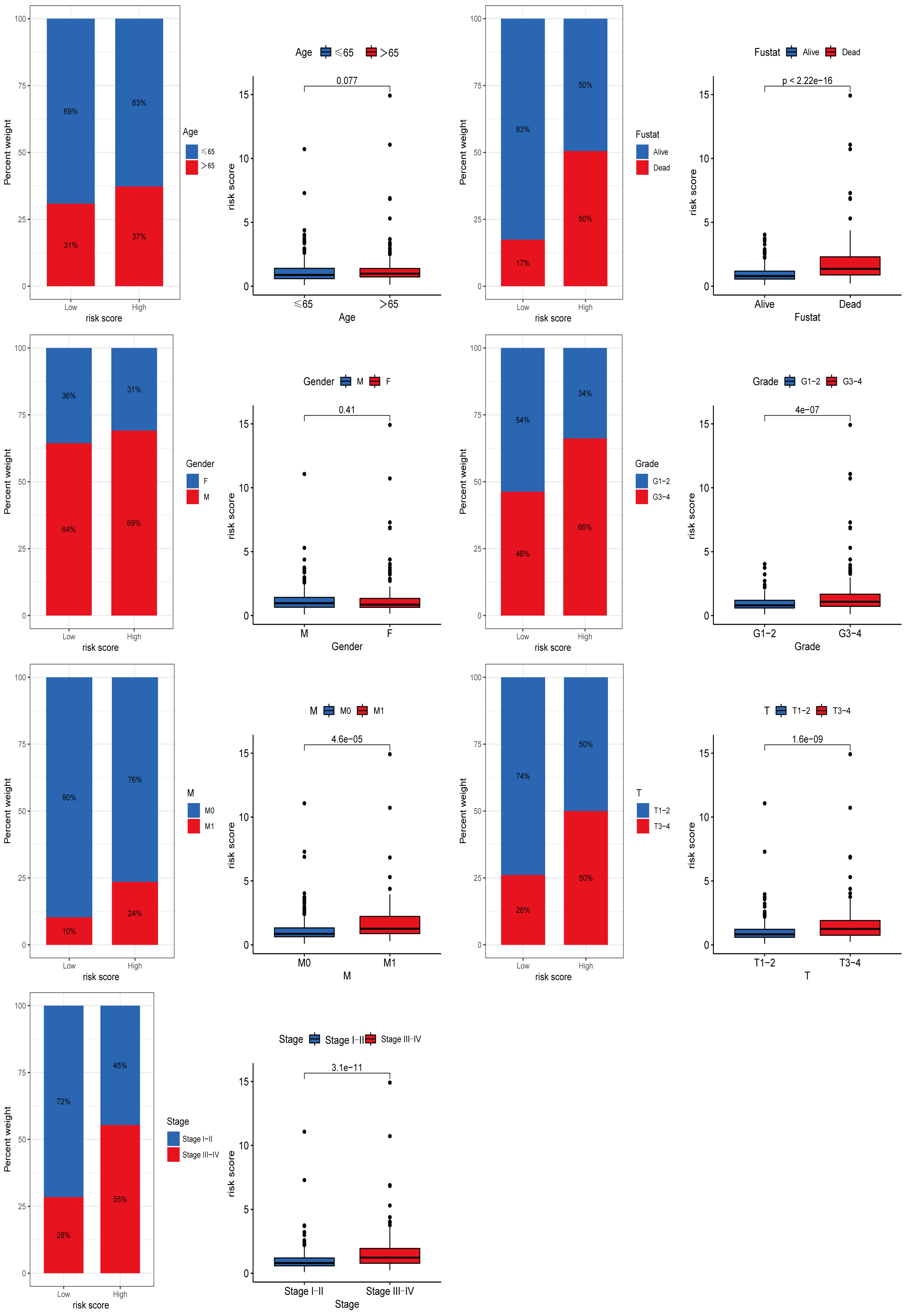


**Fig. S5.** The relationship between PRGP_score and different Clinicopathological features (including age, gender, survival status, T stage, M stage, grade and AJCC stage) in the TCGA entire cohort.


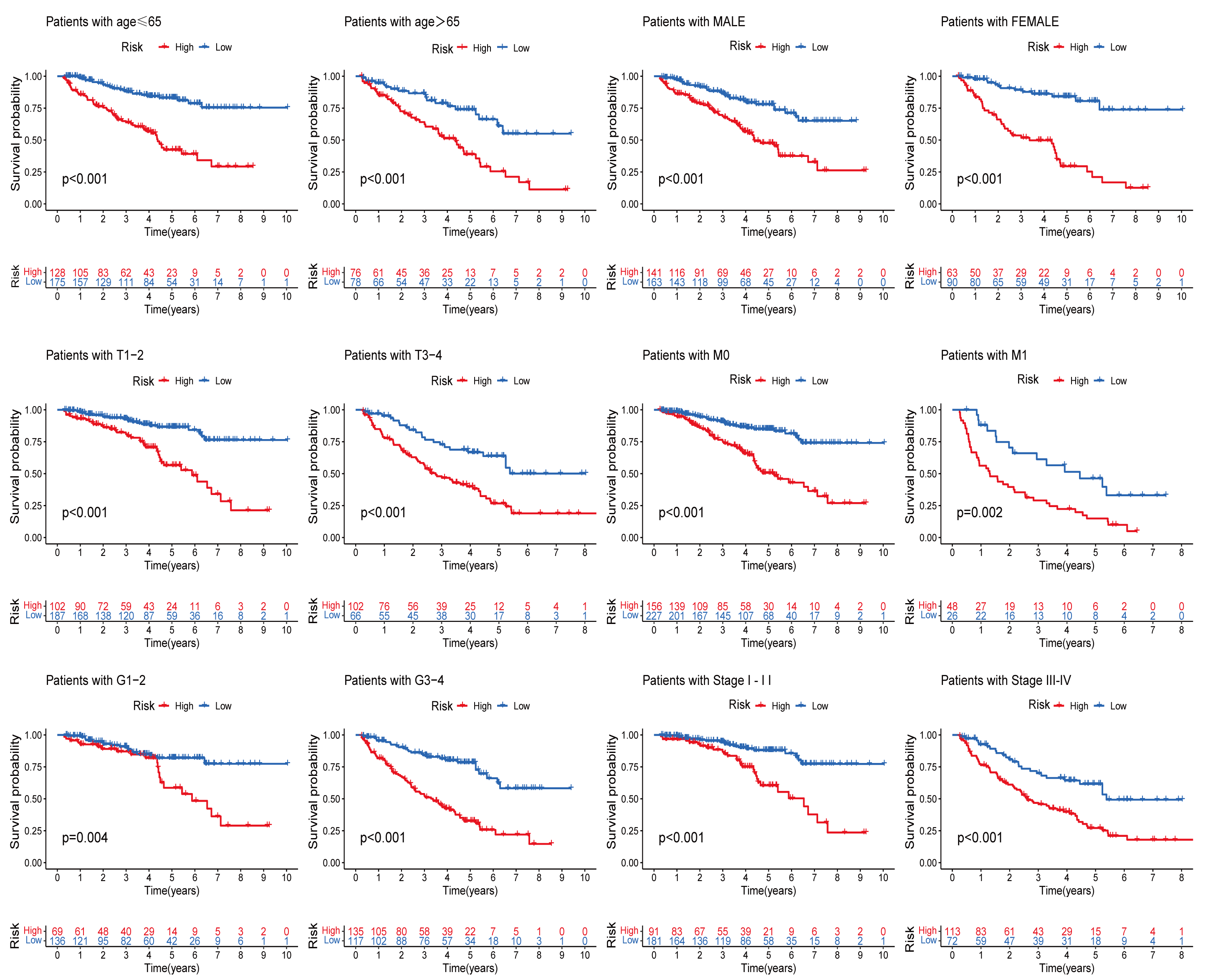


**Fig. S6.** Stratification analysis of the PRGP_score in different clinical subgroups (including age, gender, T stage, M stage, grade and AJCC stage) in the TCGA entire cohort.


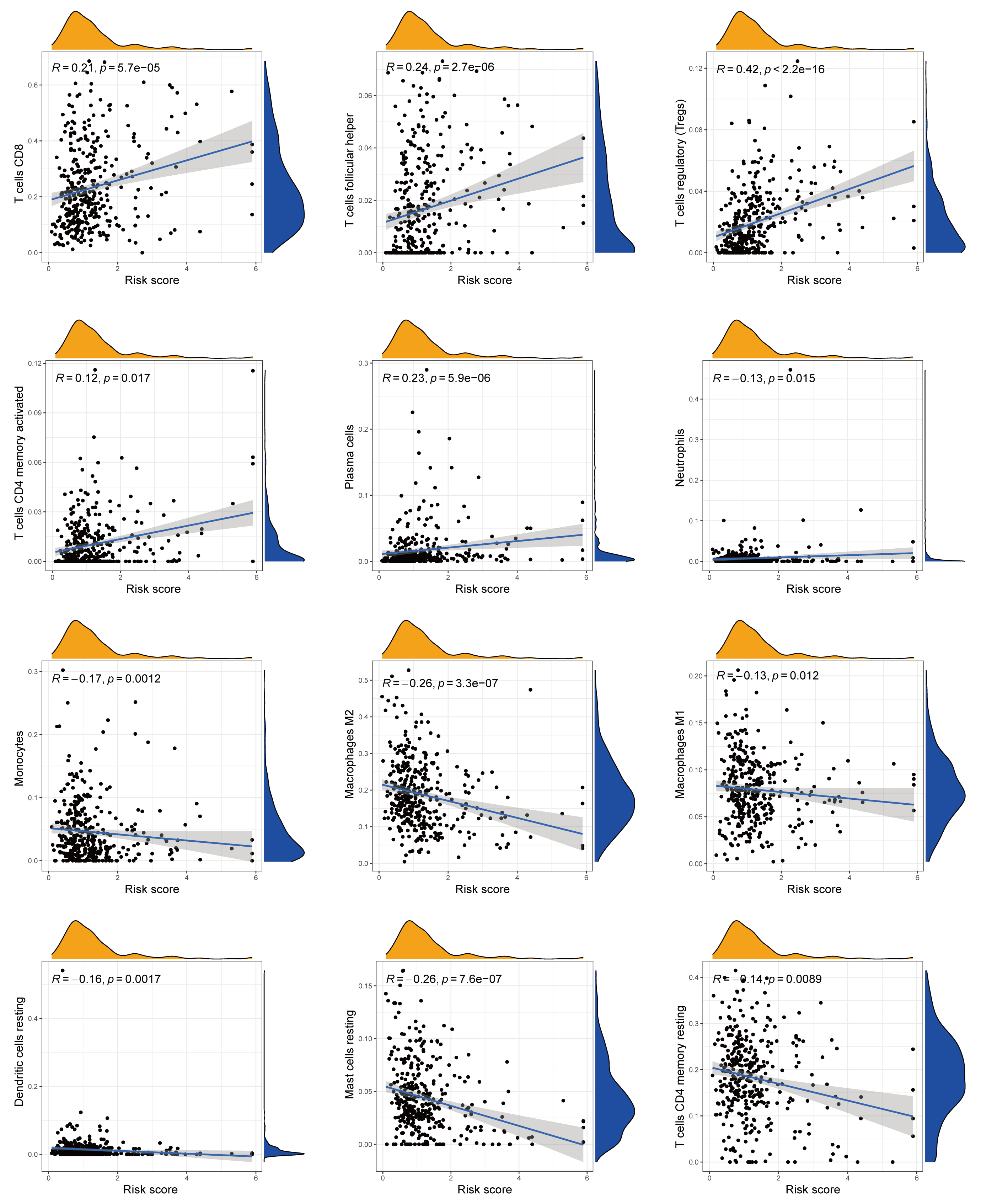


**Fig. S7.** Correlations between PRGP_score and immune cells.
